# Parahydrogen Polarization in Reverse Micelles and Application to Sensing of Protein–Ligand Binding

**DOI:** 10.1021/jacs.4c13177

**Published:** 2024-12-09

**Authors:** Pierce Pham, Oindrila Biswas, Christian Hilty

**Affiliations:** Chemistry Department, Texas A&M University, 3255 TAMU, College Station, Texas 77843, United States

## Abstract

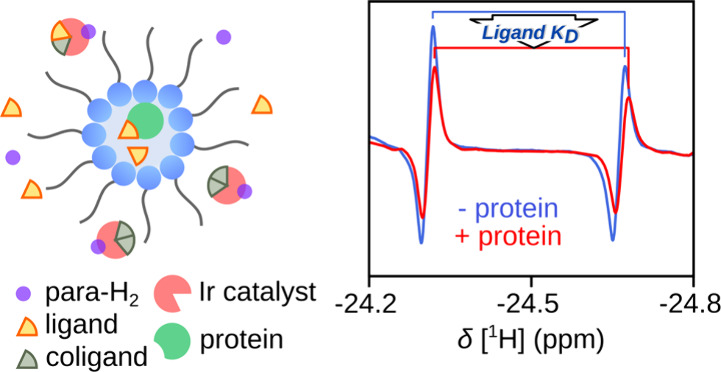

A medium containing reverse micelles supports non-hydrogenative parahydrogen induced polarization (nhPHIP) in the organic phase while solubilizing a protein in the aqueous phase. Strongly enhanced NMR signals from iridium hydride complexes report on a ligand, 4-amino-2-benzylaminopyrimidine, which crosses the phase boundary and interacts with the thiaminase protein TenA. The calculation of binding equilibria reveals a *K*_D_ of 39.7 ± 8.9 μM for protein binding. The nanoscale separation of the two phases allows the separate optimization of the parahydrogen polarization and solubilization of a biological macromolecule. The reverse micelles may be used to study other biological questions using signal enhancement by parahydrogen polarization, such as enzyme reactions, protein–protein interactions, and protein binding epitopes.

Nuclear spin hyperpolarization is enabling nontraditional applications of NMR spectroscopy: at lower concentration, in lower magnetic fields, at faster time scale, and in less pure samples. Parahydrogen induced polarization (PHIP) through hydrogenative or non-hydrogenative mechanisms has biochemical and biomedical applications including the elucidation of metabolism and magnetic resonance imaging.^1−3^ The non-hydrogenative signal amplification by reversible exchange (SABRE)^4^ technique enhances NMR signals without requiring chemical modifications.^5−7^ SABRE lends itself to the hyperpolarization of ligand molecules for measuring biomacromolecular interactions,^8−10^ along with a growing toolkit that also includes dynamic nuclear polarization,^11−13^ hyperpolarized water,^14^ and chemically induced dynamic nuclear polarization.^15^ The requirement for water as a solvent for biological molecules has posed challenges for parahydrogen polarization. Although water-soluble polarization transfer complexes exist to support SABRE or non-hydrogenative PHIP (nhPHIP),^16^ the most widely applied iridium complexes show the highest efficiency in organic solvents.

We demonstrate that a nanometer-scale dispersion of the polarization complex and protein components in a two-phase system allows parahydrogen polarization by incorporating organic solvent conditions for polarization generation simultaneously with an aqueous phase for protein solubilization (Figure 1). The organic solvent encapsulates the water droplets in reverse micelles.

**Figure 1:**
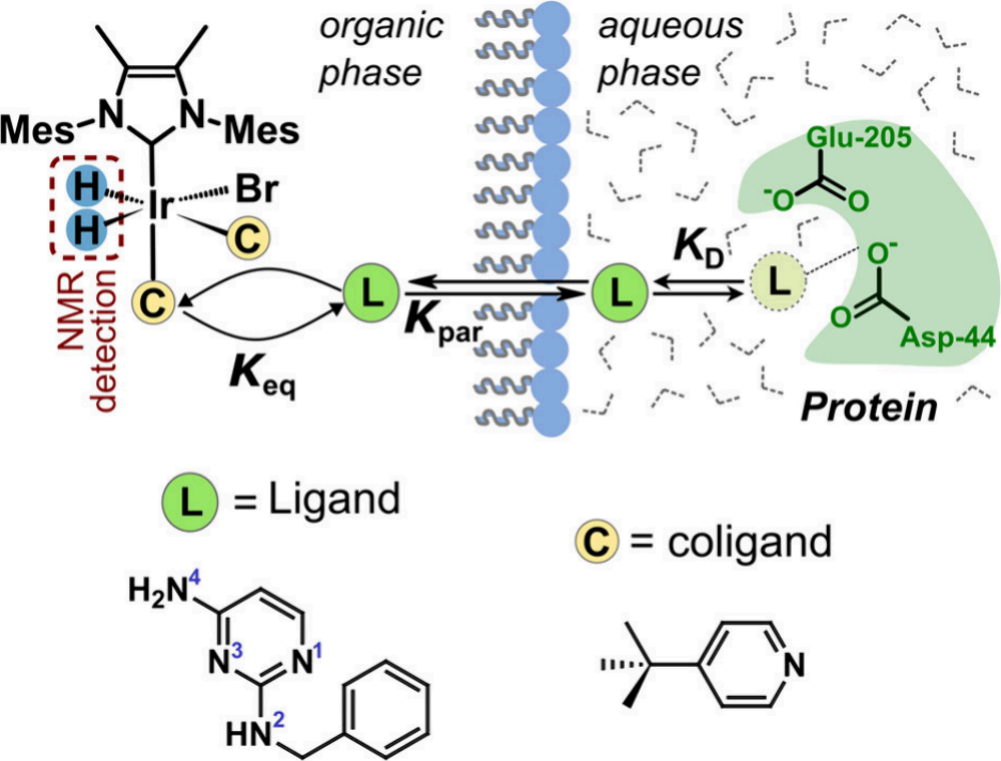
Detection of a protein–ligand interaction in reverse micelles using iridium complexes as nhPHIP sensors. Three solution equilibria are described by *K*_eq_, *K*_par_, and *K*_D_. The iridium complexes and hydrogen molecules are dominantly dissolved in the organic phase. Proteins are encapsulated in the aqueous phase. The ligand can traverse the surfactant interface to interact with the protein or iridium.

Reverse micelles have previously been exploited to improve protein structure analysis by NMR.^17−20^ Here, we measure the strongly enhanced hydride peaks of the iridium-ligand complex, instead of protein signals. The ligand molecule is in equilibrium between the organic and aqueous phases and can bind to the protein or iridium complex. The latter acts as a hyperpolarized chemosensor for detecting the presence of the ligand. In other chemosensing applications of nhPHIP, iridium hydride signals allowed detecting micromolar or submicromolar concentrations of small-molecule analytes.^21−24^

The 4-amino-2-benzylaminopyrimidine (ABAP; L) serves as the ligand (Figures 1, S1, S2). This molecule possesses a 2,4-diaminopyrimidine moiety that resembles the pyrimidine of thiamine and dihydrofolate reductase inhibitors.^25^ It binds to the 27 kDa thiaminase II (TenA), a bacterial enzyme that metabolizes thiamine, which plays a critical role in cell development and function.^26^

An nhPHIP spectrum of a reverse micelle mixture containing this ligand and a coligand for the polarization complex, 4-*tert*-butylpyridine, under equilibrium conditions, is shown in Figure 2. The ortho protons of the coligand at 8.55 ppm are enhanced 9-fold (Figures 2b and S3). Hyperpolarized signals from the ligand are not visible. The presence of the catalyst-bound ligand instead is identified through the hydride signals between −23 and −25 ppm (Figure 2c). A signal enhancement of three magnitudes (Supporting Information) is in part facilitated by the increased solubility of hydrogen in the organic solvent.

**Figure 2:**
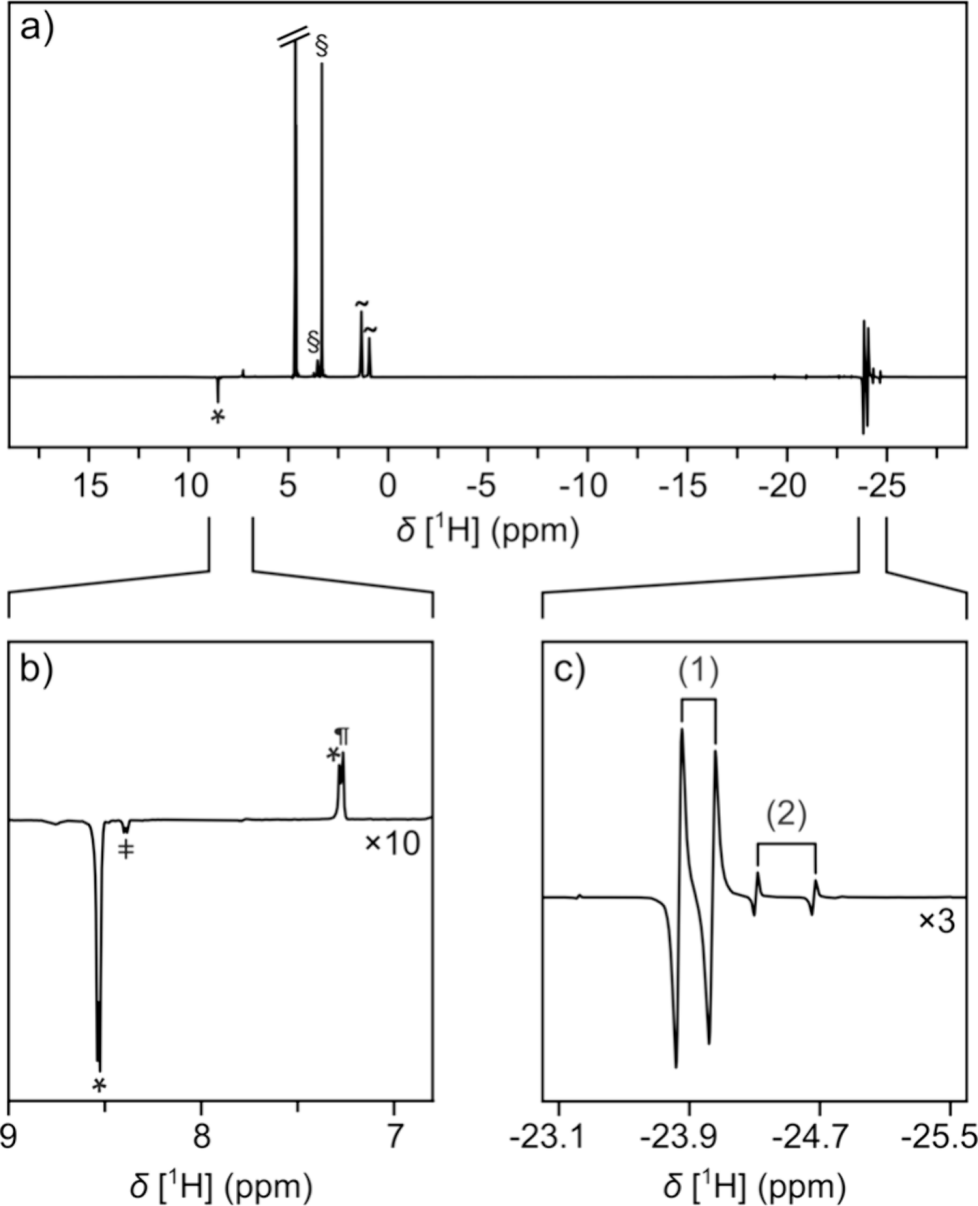
(a) Spectrum of a reverse micelle mixture measured with nhPHIP hyperpolarization. The mixture contains 100 mM cetrimonium bromide (CTAB), 100 μM ABAP ligand, 10 mM 4-*tert*-butylpyridine as coligand, and 500 μM [IrCl(*COD*)(^Me^IMes)] (^Me^IMes = 1,3-bis(2,4,6-trimethylphenyl)-4,5-dimethylimidazole-2-ylidene) in 1:1 (v/v) chloroform:heptane and 3.1% (v/v) of 50 mM Tris buffer, pH 8.0. Signals are of heptane (∼, suppressed), CTAB (§), water (4.65 ppm), and orthohydrogen (4.62 ppm; truncated). (b) Enlarged region showing the unbound coligand (*), metal-bound coligand (‡), and residual CDCl_3_ (¶) proton signals. (c) Enlarged region showing iridium hydrides of complexes (1) and (2).

Two major complexes exist in the solution (Figure 2c). The assignments of the signals are deduced from the chemical shifts of reference mixtures and from mass spectrometry (MS) (Figures 3, S4–S8). A mixture containing a coligand without a ligand results in complex (1) (Figure 3b). This complex is one of the main components in the mixture with the ligand (Figure 3c). When only the ligand is present, complex (3) is formed (Figure 3d). These signals are visible as minor components in the mixture of Figure 3c. The remaining signals in Figure 3c are attributed to complex (2), which contains both a ligand and a coligand. The isotope distributions in MS indicate the presence of bromide in all major complexes. Bromide stems from the cetrimonium bromide (CTAB) that is forming the reverse micelles and is known to complex with iridium.^27,28^ Additionally, iridium dimers were identified in MS, which may have formed during solvent evaporation (Figure S8). The hydride region in the NMR spectra in Figure 2 contains large signals only for complexes (1) and (2), indicating that other species, including dimers, are not significantly populated in the solution. The formation of the complexes can be observed as a function of time after the sample is pressurized with parahydrogen (Figure S9). The time dependence follows a typical activation behavior observed for SABRE catalysts.^29^

**Figure 3:**
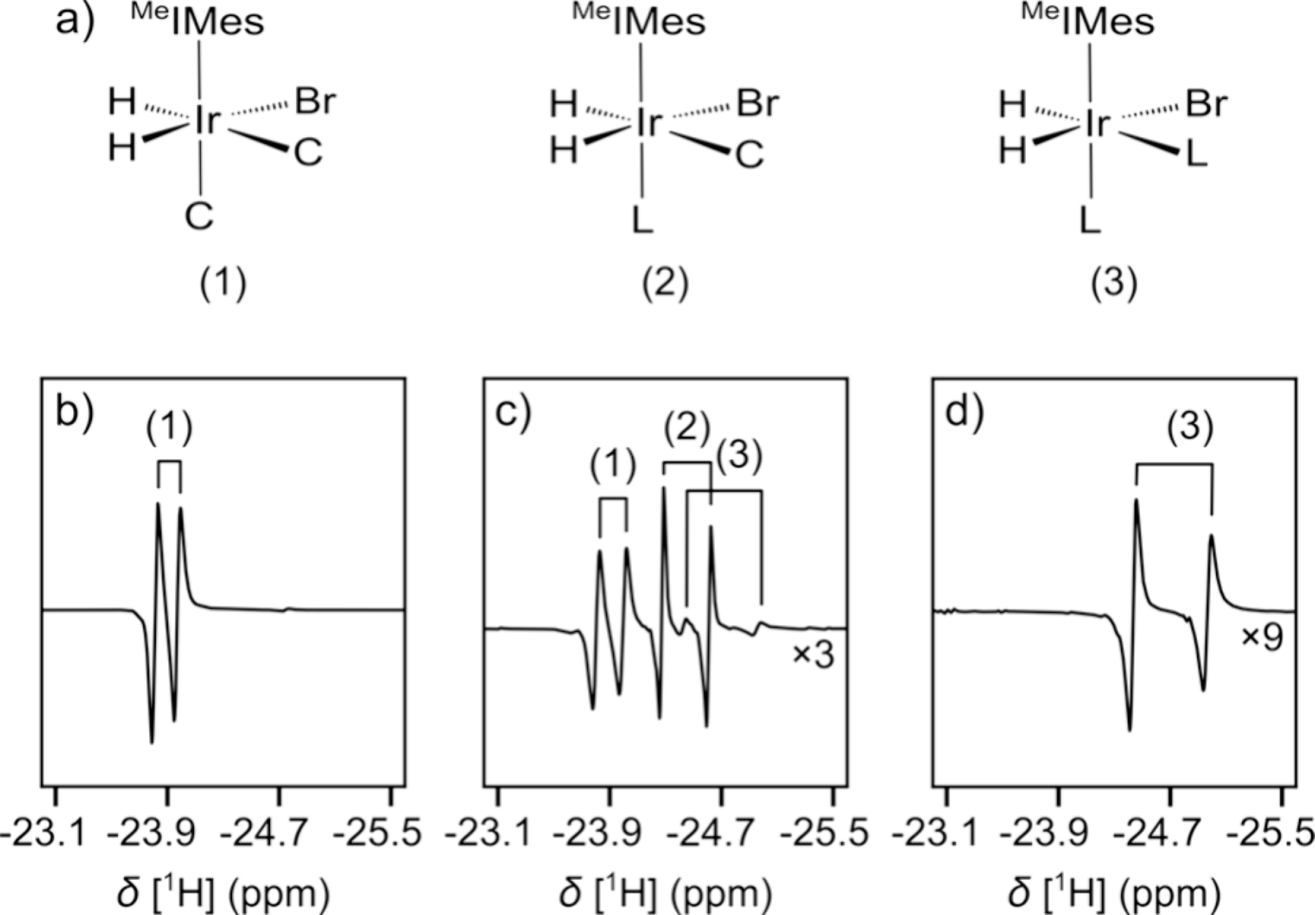
(a) Structures of iridium complexes (1)–(3), comprising ligand L and coligand C. (b) Hydride region of the NMR spectrum of 500 μM Ir(^Me^IMes)Cl(*COD*) and 10 mM coligand in reverse micelles. (c) Spectrum of a mixture as in (b), additionally containing 1 mM ligand. (d) Spectrum as in (c), in the absence of a coligand. Numbers identify the complexes in the spectra.

The fact that no SABRE enhancement was observed for the ABAP ligand, while a 9-fold enhancement was detected for the coligand, suggests that ABAP binds in the axial position of the octahedral complex, where polarization is generally not observed.^30^ This binding mode is consistent with the higher steric hindrance at the trans sites, which may not accommodate the bulk of the ABAP ligand. Likely, N1 of ABAP is binding to iridium, with N3 being more hindered due to two ortho amino groups.

A spectrum of a reverse micelle solution with protein is shown in Figure S10 for comparison with Figure 2. A difference in the relative intensity of the iridium hydride signals from complexes (1) and (2) is observed with and without TenA protein (Figures 4a–d and S11, S12, Tables S1, S2). This change is due to a shift in the equilibrium of the ABAP ligand partitioning in the aqueous and organic phases when the ligand is binding to the protein. It is quantified by the signal ratios *R*_H_ in Figures 4e,f. In the data set shown, this ratio reduces from *R*_H_ = 0.0313 to *R*_H_ = 0.0215 upon addition of the protein.

**Figure 4:**
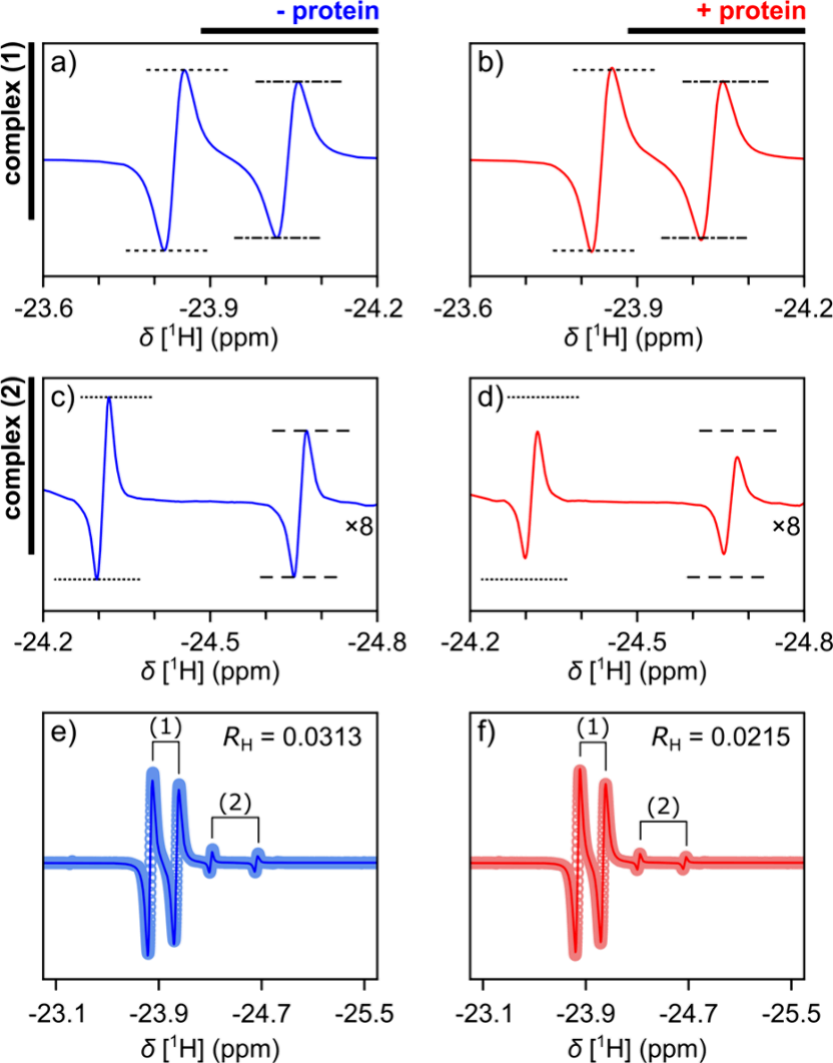
(a–d) nhPHIP signals of complexes (1) and (2) in the absence and presence of an overall 102 μM TenA protein. Horizontal lines are drawn at equal positions to compare the signal intensities. (e, f) Fitted Lorentzian functions (−) and data points of the spectra (○) in the absence and presence of protein. The ratios of the fitted signal integrals from complex (2) divided by complex (1) are indicated as *R*_H_. (a), (c), (e) and (b), (d), (f) are from the same spectrum, respectively.

The reverse micelles exhibited a diameter of 10 nm (Figure S13 and Table S3) and constituted 3.1% of the sample volume. The 100 mM CTAB is above the critical micelle concentration of 40 mM reported in a similar solution.^31,32^

In the following, the calculation of the protein–ligand binding affinity is demonstrated from a quantitative analysis of the change in the concentration ratio of the two complexes in the organic phase,
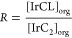
1

The resulting equations allow the determination of the dissociation constant *K*_D_ for the protein and ligand based on the assumption that the concentration ratio is equal to the ratio *R*_H_ of signal integrals. This assumption would be generally accepted in conventional NMR spectra but is not trivial here because of the possibility of differing hyperpolarization efficiencies and relaxation effects. The validity of the assumption is further substantiated below.

We consider three equilibrium processes based on Figure 1,
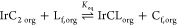
2
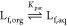
3
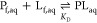
4which include the ligand L, coligand C, complex (1) as IrC_2_, complex (2) as IrCL, the protein P, and the protein–ligand complex PL. Subscripts indicate unbound, i.e., free species (f), and location in aqueous (aq) or organic (org) phases.

Using the equilibrium equations, first, *K*_eq_ can be calculated from *R*_H_ measured without protein (eq S18). The partition coefficient *K*_par_ = 0.216 ± 0.028 is separately determined (eq S29). Second, with knowledge of *K*_eq_, the *K*_D_ can be found from the corresponding *R*_H_ measured with protein (eqs S20–S22, S8). Based on the shift of the equilibrium, the calculation of *K*_D_ requires only two data sets, measured with and without protein. A titration that would be employed with parameters such as *R*_2_ relaxation or chemical shift, which have an unknown end point, is not required.

In three sets of the two experiments, mean *R*_H_ values are 0.0297 ± 0.0005 and 0.0216 ± 0.0002 in the absence and presence of TenA protein, respectively (Tables S1, S2). The significant decrease in the measured values upon the addition of protein forms the basis for the calculation of *K*_D_. The standard deviations of *R*_H_ obtained for each sample are 3% or less, indicating the reproducibility of the NMR measurement and Lorentzian fitting. The results lead to a *K*_eq_ value of 3.16 ± 0.29 for the binding of a ligand to the iridium catalyst complex. By including the data measured with protein, the *K*_D_ of the ABAP ligand binding to TenA is found as 39.7 ± 8.9 μM. This result is from Monte Carlo simulations (Figures S14, S15, Table S4). The *K*_D_ value is in excellent agreement with 39.8 ± 6.9 μM obtained from a titration of TenA with ABAP (Figures S16, S17). Simulations of the binding equilibria indicate that the range of *K*_D_ values to which the experiment is sensitive depends on *K*_par_ (Figure S18), pointing to a mechanism for optimizing the experiment for different protein–ligand systems.

The dependence of *R*_H_ on the ligand concentration is seen in a control titration using the same setup (Figures S19, S20, Tables S5, S6). The *K*_eq_ = 3.15 ± 0.05 fitted from the titration (eq S26) agrees within error ranges with the values from the experiment (Table S4). The self-consistency of the data at multiple concentrations shown in the titration supports the above assumption that the signal ratios can be used for the concentration ratios in the analysis.

The use of the ratio of hydride signals of complexes (2) to (1), rather than signals of complex (2) alone, increases the accuracy. The amplitudes of complexes (1) and (2) fluctuated by 13–16% in the reference titration (Table S5). Contributing factors may include the flow rate and pressure of hydrogen. Conversely, the spread of the corresponding *R*_H_ values is only ∼5% (Table S6).

The micromolar range concentration of the ligand demonstrates the benefits of nhPHIP in reducing the limit of detection. At a ligand concentration of 1 μM, resulting in 0.12 μM complex (2), the signals of the complex are still observable in a single scan in the 400 MHz NMR spectrometer (Figure S21). These concentrations compare favorably to typical ranges of 25–100 μM in ligand binding studies using conventional NMR, which employs signal averaging.^33^ The nhPHIP experiments were performed in a single scan but would also be compatible with signal averaging to increase the signal-to-noise ratio.

The determination of the dissociation constant of a protein and ligand is demonstrated as one application of nhPHIP in a two-phase reverse micelle system. The experiment foremost requires a ligand that is soluble in both phases and exhibits an observable change in the experimentally obtained *R*_H_ when the protein is added. These changes can be tuned by choosing a suitable coligand^34^ or catalyst.^35^ A purpose-designed ligand with the required properties could further be used as a reporter for the screening of other putative ligands to the protein in a binding competition.^10^

The nhPHIP experiment is a new option for the detection of *K*_D_ using spin hyperpolarization, alongside methods such as D-DNP^36^ and photo-CIDNP.^15^ Similar to photo-CIDNP, it makes use of a single-pot reaction mixture, where scans, in principle, can be repeated for signal averaging.

Reverse micelles with nhPHIP could be exploited for other biological applications. Iridium sensors may be used to probe biological products of enzymatic reactions. The expected submicromolar sensitivity approaches that of UV methods, but NMR chemical shifts also differentiate individual products with similar structures. Another range of applications can be based on direct observation of hyperpolarized molecules. A ligand hyperpolarized by SABRE in the organic phase can diffuse to the aqueous phase, where its interaction with a protein can be observed. To hyperpolarize the ligand, the iridium complexes should be optimized for the required complex lifetime.

A hyperpolarized ligand would further transfer its polarization to a protein via cross relaxation, potentially enabling studies of the protein binding epitope structure or protein–protein interactions. The quality of protein spectra would further benefit from the lowered rotational correlation time (*τ*_c_) of the macromolecules in the reverse micelles.^17^

In conclusion, we demonstrated that the nanoscale dispersion in a CTAB/chloroform/heptane reverse micelle system can solubilize an iridium catalyst for parahydrogen polarization in its organic phase and encapsulate a protein in the aqueous phase. A ligand for the protein can partition between the phases and interact with both the catalyst and the protein. The comparison of iridium hydride signals between the experiments with and without the protein allows an accurate determination of the binding affinity of the ligand. Other applications may further involve polarization transfer to the aqueous phase of the reverse micelles.

## Supporting Information Available

Experimental procedure, synthesis, and characterization of 4-amino-2-benzylaminopyrimidine, identification of iridium complexes, data fitting, activation of precatalysts, effect of protein on the hydride signals, ligand binding experiments, dynamic light scattering measurements, equilibrium calculations, enzymatic assay, simulation of change in *R*_H_ values, dependence of hydride signal intensity on ligand concentration, detection limit, determination of organic/aqueous partition coefficient *K*_par_, protein expression and purification (PDF)
