# Supplementary material for: Parahydrogen Polarization in Reverse Micelles and Application to Sensing of Protein–Ligand Binding
Source: J Am Chem Soc. 2024 Dec 9;146(50):34274–8. doi: 10.1021/jacs.4c13177 (PMC11664502; doi:10.1021/jacs.4c13177)
Supplement: Supplementary file 1 — ja4c13177_si_001.pdf [file ja4c13177_si_001.pdf]

Supporting Information

Parahydrogen Polarization in Reverse Micelles and Application to Sensing of Protein-Ligand Binding

Pierce Pham[#], Oindrila Biswas[#] and Christian Hilty*

Chemistry Department, Texas A&M University, College Station, TX 77843, USA

[#]equal contribution

*email: chilty@tamu.edu

Table of Contents

Experimental Procedure.....2
Synthesis and Characterization of 4-amino-2-benzylaminopyrimidine.....2
Characterization Methods.....2
Synthesis.....3
Identification of Iridium Complexes.....5
Data Fitting.....8
Activation of Precatalysts.....9
Effect of Protein on the Hydride Signals.....9
Ligand Binding Experiments.....10
Dynamic Light Scattering Measurements.....14
Equilibrium Calculations.....15
Calculation of K_{eq} from an Experiment without Protein.....16
Calculation of K_D from an Experiment with Protein and using K_{eq}.....17
Experimental Values.....17
Enzymatic Assay.....20
Simulation of Change in R_H values.....21
Dependence of Hydride Signal Intensity on Ligand Concentration.....22
Detection Limit.....25
Determination of Organic/Aqueous Partition Coefficient K_{par}.....26
Protein Expression and Purification.....26
References.....27

Experimental Procedure

Stock solutions of 500 mM CTAB (TCI, Portland, OR), of 5 mM Ir(^{Me}IMes)(COD)Cl (Strem, Newburyport, MA) as the precatalyst, of 100 mM 4-tert-butylpyridine (Sigma-Alrich, St. Louis, MO) as the coligand, and of 2 mM 4-amino-2-benzylaminopyrimidine (ABAP; synthesis in the later section) as the ligand were prepared. All stock solutions were in $CDCl_3$ (Cambridge Isotope Laboratories, Andover, MA). COD is 1,5-cyclooctadiene. ^{Me}IMes is [4,5-dimethyl-1,3-bis(2,4,6-trimethylphenyl)imidazol-2-ylidene. To prepare samples, 200, 100, 100, and 50 µL of the stocks of CTAB, precatalyst, coligand, and ligand solutions were added consecutively to a volume of 485 µL heptane, and mixed well. Then, 30 µL of Tris buffer, pH 8.0 or 3.4 mM of thiaminase II (synthesis and purification in the later section) in the same buffer was added to the previous mixture in one portion. This mixture was vortexed for 15 seconds and left to equilibrate for about 1 minute. During this time, the solution turned from slightly cloudy to clear. In this procedure, the volume of the aqueous phase was optimized to obtain clear solutions, which was achieved at 3.1% (v/v). Clear solutions were not observed when the volume of the aqueous buffer added was too large. A 500 µL portion of this mixture was transferred to an NMR tube. To activate the precatalyst, this sample was pressurized with 120 psi of hydrogen gas for 20 minutes. The hydrogen gas was enriched to 95% parahydrogen using a cryogenic system (Advanced Research Systems, Macungie, PA) operating at a temperature of 29 K. The hydrogen gas pressure was maintained during all experiments with the sample. For NMR experiments with parahydrogen polarization, the sample was placed in a 9.4 T magnetic field of an NMR spectrometer. Parahydrogen gas at the same pressure was bubbled through the sample with a flow rate of 0.2 standard liters per minute for 5 seconds and stabilized for 1 second before applying NMR pulses. Heptane NMR signals were suppressed by selective excitation with repeated EBURP2-shaped $\pi/2$ pulses at 1.14 ppm for 5 ms and dephasing by alternative, randomized pulsed-field gradients G_z, G_y, and G_x for < 1 ms. Free induction decays (FIDs) were acquired after a $\pi/4$ hard pulse. Exponential window functions with a line broadening of 1 Hz were applied to the FIDs before Fourier transform to obtain frequency spectra. Data was further processed by fitting Lorentzian functions to the signals, as described later.

Synthesis and Characterization of 4-amino-2-benzylaminopyrimidine

Characterization Methods

NMR data was acquired using a Bruker 400 MHz NMR spectrometer (^{1}H at 400 MHz and ^{13}C{^{1}H} at 100 MHz) equipped with a broad-band probe. Spectra were processed with Bruker Topspin software, version 4.1.1. Multiplicity was indicated as: s (singlet), d (doublet), and multiplet (m). All the coupling constants are reported in Hertz (Hz).

High resolution mass spectra were measured by the Laboratory for Biological Mass Spectrometry at Texas A&M University, College Station, TX 77840. Electrospray ionization mass spectrometry (ESI-MS) experiments were performed using a Thermo Scientific Q Exactive Focus mass spectrometer.

Synthesis

The synthesis of 4-amino-2-benzylaminopyrimidine (ABAP) as a TenA ligand is summarized in the following procedure. A sealed 5-mL flask was charged with a PTFE-coated stir bar, 0.65 g of 4-amino-2-chloropyrimidine (TCI, Portland, OR), and 1.1 g of benzylamine (TCI). This mixture was heated at 100 ºC with stirring for 2 days and then cooled to room temperature. The mixture was subsequently added to 50 mL of boiling water with continuous stirring for a few minutes. After that, this mixture was cooled down to room temperature. The white solid was filtered and dried under vacuum. The product yield was 0.62 g or 62%.

^{1}H NMR δ (ppm) (400 MHz, $CDCl_3$): 7.89 (d $^{3}J_{HH}$ = 5.5, 1H, pyrimidine), 7.39-7.23 (m, 5H, phenyl), 5.81 (d, $^{3}J_{HH}$ = 5.6, 1H, pyrimidine), 5.42 (bs, 1H, NH), 4.70 (bs, 2H, NH_2), and 4.62 (d, $^{3}J_{HH}$ = 5.8, 2H, CH_2).

^{13}C NMR δ (ppm) (100 MHz, $CDCl_3$): 163.6, 162.4, 156.9, 139.6, 128.5, 127.4, 127.1, 95.6, and 45.2.

Positive ESI-HRMS M/Z: found 201.1131, calculated 201.1135 for $[C_{11}H_{13}N_4]^+$ as $[M+H]^+$

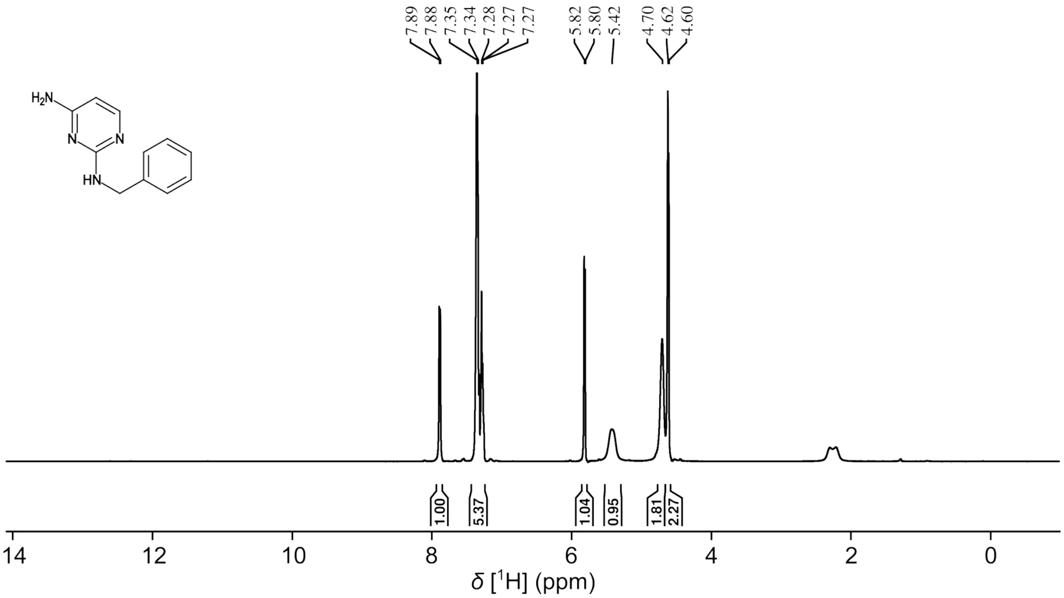

Figure S1: *^{1}H NMR spectrum of 4-amino-2-benzylaminopyrimidine (ABAP) in $CDCl_3$.*

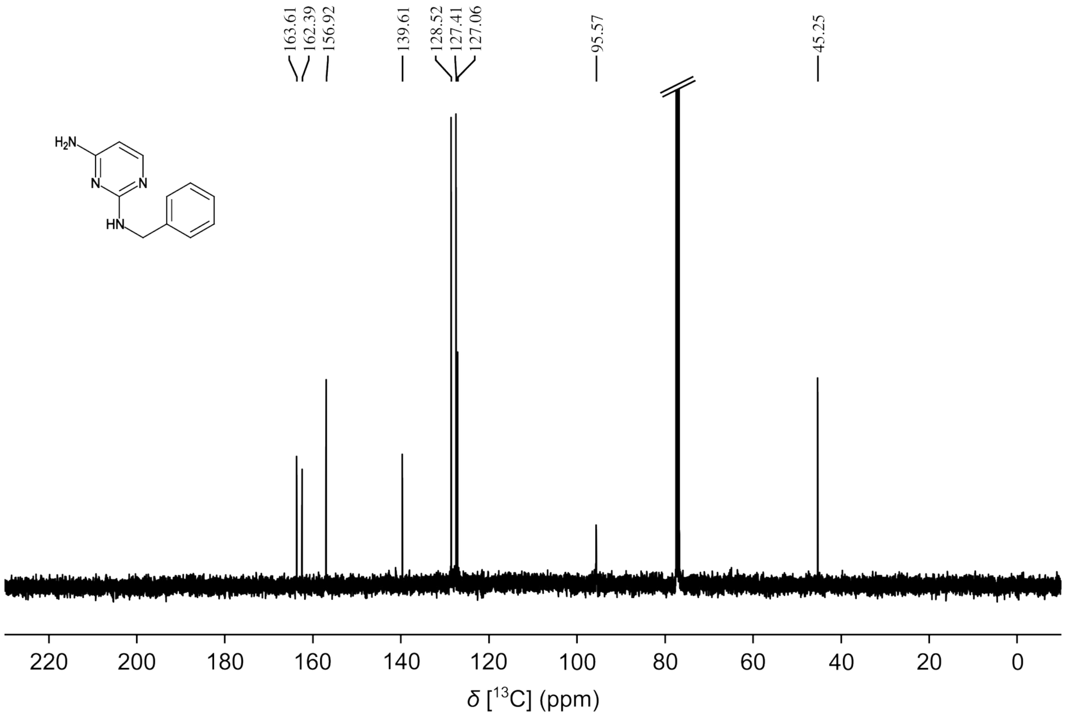

Figure S2: *^{13}C NMR spectrum of 4-amino-2-benzylaminopyrimidine (ABAP) in $CDCl_3$. The dashed line represents the truncated solvent peak centered at 77.06 ppm.*

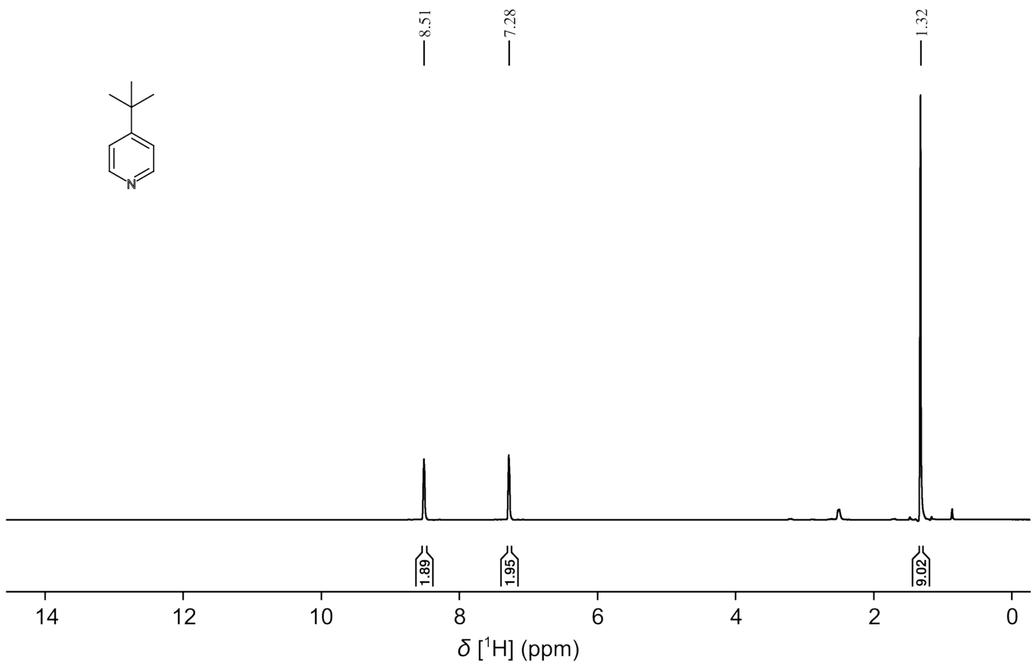

Figure S3: *^{1}H NMR spectrum of 4-tert-butylpyridine in $CDCl_3$.*

^{1}H NMR δ (ppm) (400 MHz, $CDCl_3$): 8.54 – 8.48 (m, 2H, phenyl), 7.31 – 7.24 (m, 2H, phenyl), 1.32 (s, 9H).

Identification of Iridium Complexes

The components of reverse micelles are readily visible in the spectrum (Figure 2). Peaks include suppressed heptane signals at 1.35 and 0.95 ppm, CTAB signals at 3.54 and 3.33 ppm, water signal at 4.65 ppm, and orthohydrogen signal at 4.62 ppm. The high concentrations of the coligand and catalyst were chosen to drive the chemical equilibrium forming octahedral catalyst complexes and lessen the contribution of other catalyst complexes with lower coordination numbers. The parahydrogen enhances the hydride NMR signals of Ir(^{Me}IMes)H_2XBr complex, visible as antiphase signals from -23.5 to -25 ppm, where X can be C_2 for complex (1) and can be CL in case of complex (2) as described in the main text. In aprotic solvents such as the organic phase of the reverse micelles, the lack of ionic solvation causes anions to stay bound to iridium centers and cis to NHC ligands such as ^{Me}IMes.[1] This leads to the formation of asymmetric structures of iridium complexes.

To identify the chemical shifts arising from different iridium complexes, spectra were acquired from reverse micelle solutions in the presence of the ligand and coligand after parahydrogen bubbling (Figure 3c). Spectra were acquired for the reverse micelle solution in the absence of a ligand for complex (1) (Figure 3b) and in the absence of a coligand for complex (3) (Figure 3d). The peaks for complex (1) at -23.8 and -24.0 ppm and complex (2) at -24.3 and -24.7 ppm (Figure 3c) were assigned based on the peaks of complex (1) at -23.8 and -24.0 ppm (Figure 3b) and of complex (3) at -24.4 and -25.0 ppm (Figure 3d). The chemical shifts were referenced to the $CDCl_3$ signal at 7.28 ppm.

The iridium hydride signal enhancement was calculated by comparing fitted Lorentzian peaks of the hydride signals (described below) with those of the $(NCH_3)_3$ peak of CTAB at 3.33 ppm. The signal enhancement is

$$\epsilon = \frac{A_{\text{complex}} \cdot C_{\text{CTAB}} \cdot 9}{A_{\text{CTAB}} \cdot C_{\text{complex}} \cdot 2} \tag{S1}$$

A_{complex} is the area obtained for the hydride signals of complex 1 or 2 tabulated in Table S1, C_{CTAB} = 100 ± 5 mM is the concentration of CTAB, A_{CTAB} is the area for the $(N(CH_3)_3)$ peak of CTAB and C_{complex} is the concentration of complex 1 or 2 as obtained from Eqs. S14 and S15. The 2 and 9 are the numbers of protons for the iridium hydride and CTAB signals, respectively. From the data in Figure 2, the resulting signal enhancement value is ϵ = 9217 ± 660. This value is understood as the nominal signal enhancement prior to any cancellation of the positive and negative parts of the hydride signals.

Electrospray ionization mass spectra of the reverse micelle solutions were performed to further identify the asymmetric iridium complexes formed in the nhPHIP reaction mixtures. Solutions containing reverse micelle were prepared according to the procedure described above with CTAB (100 mM), ligand ABAP (1 mM), coligand (10 mM), iridium precatalyst (2 mM), and 3% buffer fraction in $CDCl_3$/heptane. After performing nhPHIP with the sample, an ESI mass spectrum of the sample was acquired by diluting the sample 100 times with MeOH (Figure S4). In Figure S4, the highest intensity signals appear at m/z = 284.3303 and at m/z = 649.5773. These signals are identified as $[\text{CTAB-Br}]^+$ and $[(\text{CTAB-Br})_2\text{+Br}]^+$, respectively. A peak at m/z = 940.3059 was found to be of complex (2)-H_2, as seen in Figure S5. In the same Figure S5, a peak at m/z = 860.3952 is observed, which corresponds to the species [Ir(^{Me}IMes)(L)(C)Br-2H-Br], where L and C stand for ligand and coligand, respectively. A similar solution was prepared without adding ABAP ligand (Figure S6). Similarly, when the solution

was prepared in the absence of the ligand, a peak at m/z = 873.3043 was identified due to the activated iridium complex [Ir(^{Me}IMes)(C)$_2$Br], complex (1), as seen in Figure S7. Other signals of the different activated iridium complexes were also found in this experimental condition. These signals are labeled as identified in Figure S7. Peaks at m/z > 1000 were further seen in the ESI mass spectra (Figure S8), and are attributed to dimeric iridium complexes.

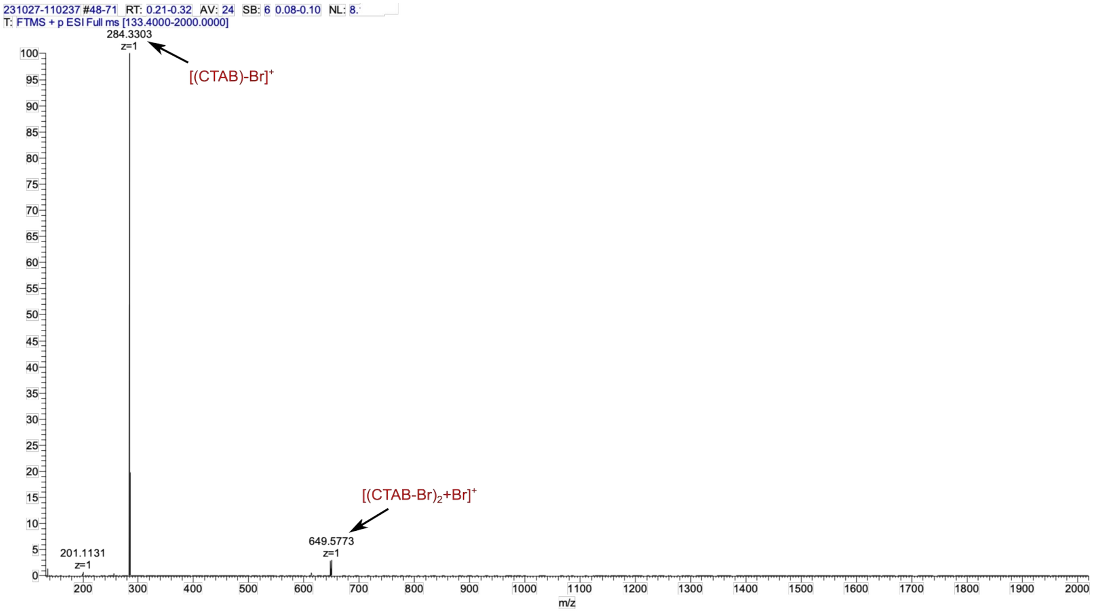

Figure S4. *Positive ESI mass spectrum of activated Iridium complex (0.5 mM), 100 mM CTAB, 1 mM ligand, 10 mM coligand, and 3% buffer in $CDCl_3$/heptane. The ESI-MS spectrum was recorded after nhPHIP.*

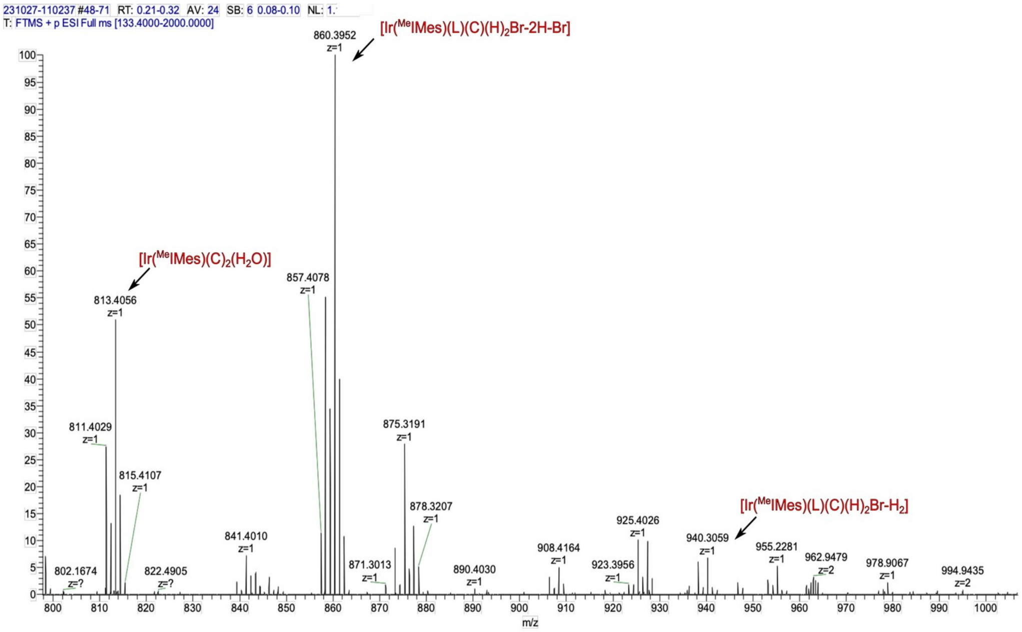

Figure S5. *Zoomed-in ESI-MS spectrum from Figure S4 showing m/z=800…1000. L and C in the figure stand for ligand and coligand, respectively.*

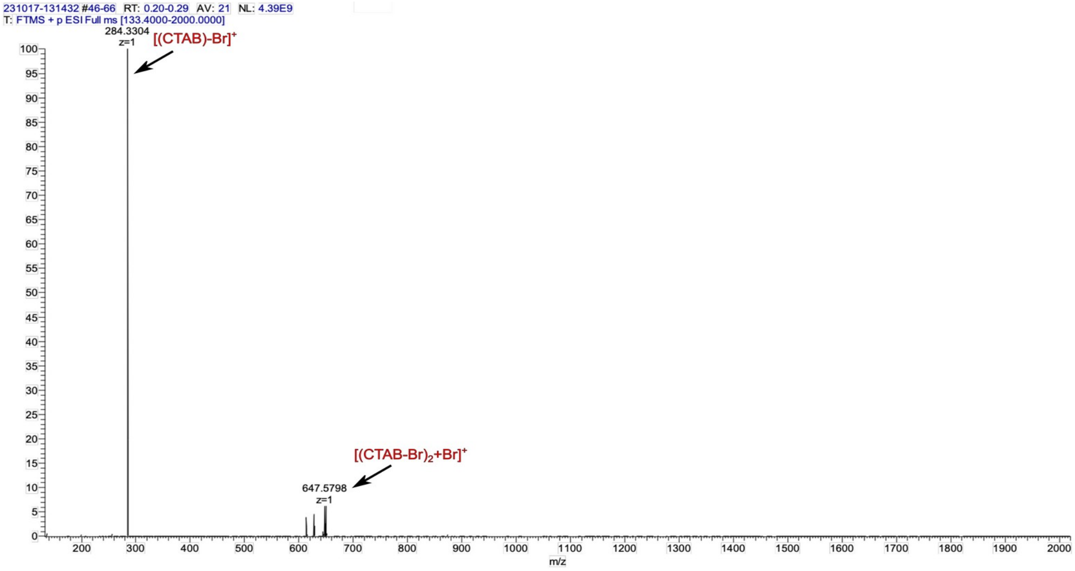

Figure S6. *Positive ESI mass spectrum of reverse micelle solution as in Figure S4 in the absence of the ligand in the reverse-micelle solution (iridium precatalyst 0.5 mM, 100 mM CTAB, 10 mM coligand, and 3% buffer in $CDCl_3$/heptane). The ESI-MS spectrum was recorded after nhPHIP.*

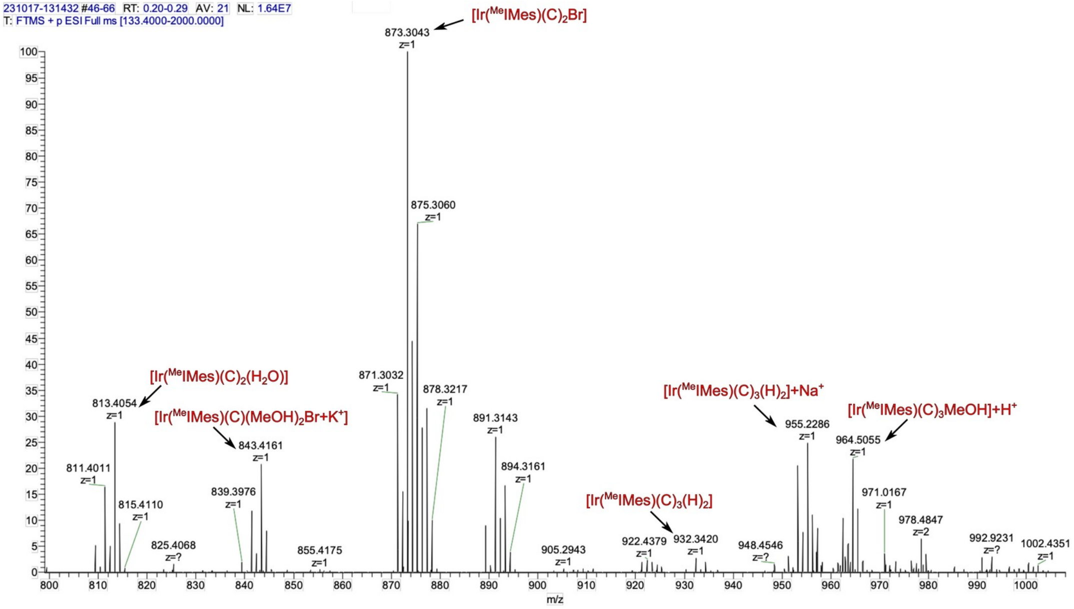

Figure S7. *Zoomed-in ESI-MS spectra from Figure S6 showing m/z=800…1000. C in the figure stands for coligand.*

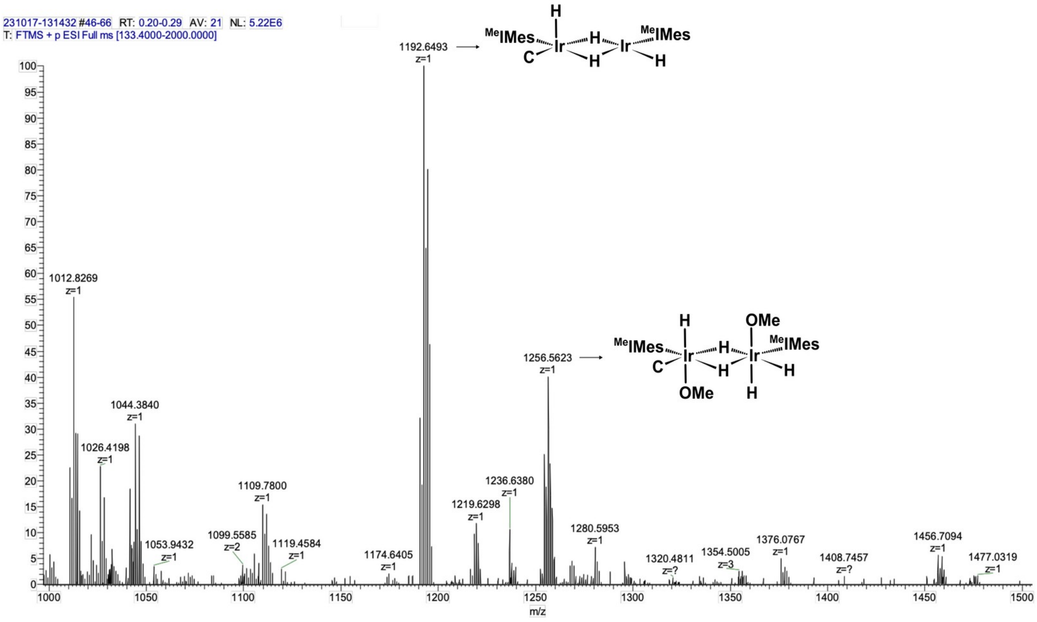

Figure S8. *Zoomed-in ESI-MS spectra from Figure S6 showing m/z=1000…1500 showing signals assigned to dimers. C stand for coligand.*

Data Fitting

Individual signals centered at -23.8, -24.0, -24.3, and -24.7 ppm were fitted with Lorentzian functions

$$L(w)=\frac{Ar}{r^2+(w-w_p)^2} \quad \text{(S2)}$$

where A is the amplitude and r is a parameter related to the peak width. The position of each peak is $w_p = (w_0 \pm J/2)$, with w_0 the center frequency of each iridium hydride signal. J is the coupling constant of the two hydride protons, which was set to 7.2 Hz for the fit. This value was determined experimentally from the splitting of the hydride peaks of complex 1 and complex 2 observed at 277 K using non-*para* enriched hydrogen and is in agreement with the zero-crossing of signals in a Singlet-to-Magnetization, S2M pulse sequence (π/4-τ- π- τ, τ=1/4*J*).[2] Each signal included two Lorentzian lines with two amplitudes, two central frequencies of (w_o – J/2) and (w_o + J/2), and one peak width (r). The fit parameters for the Lorenztian function are the four width-related parameters (r_1, r_2, r_3, and r_4) for four iridium hydride peaks and eight amplitudes, namely, $A_{1,\mathrm{IrCL},1}$, $A_{1,\mathrm{IrCL},2}$, $A_{2,\mathrm{IrCL},1}$, $A_{2,\mathrm{IrCL},2}$, $A_{1,\mathrm{IrC2},1}$, $A_{1,\mathrm{IrC2},2}$, $A_{2,\mathrm{IrC2},1}$, $A_{2,\mathrm{IrC2},2}$.

$A_{1,\mathrm{IrCL}}$, $A_{2,\mathrm{IrCL}}$, $A_{1,\mathrm{IrC2}}$, and $A_{2,\mathrm{IrC2}}$ are the amplitude values for the peaks centered at -23.8, -24.0, -24.3, and -24.7 ppm, respectively.

The amplitudes for the hydride peaks can be represented as follows,

$$A_{1,IrX}=A_{1,IrX,1}+A_{1,IrX,2} \quad \text{(S3)}$$

$$A_{2,IrX}=A_{2,IrX,1}+A_{2,IrX,2} \quad \text{(S4)}$$

where X is C_2 for the peaks centered at -23.8 and -24.0 ppm, and CL for the peaks centered at -24.3 and -24.7.

From the fitted parameters, the ratios of signal intensities from the iridium complexes of Ir(^{Me}IMes)H_2C_2Br and Ir(^{Me}IMes)H_2(L)(C)Br, abbreviated as complex (1) and complex (2) respectively, were calculated as

$$R_H = \frac{A_{1,IrCL} + A_{2,IrCL}}{A_{1,IrC2} + A_{2,IrC2}} \quad \text{(S5)}$$

Activation of Precatalysts

The samples that were described in the experimental procedures section were activated by pressurization with 120 psi of parahydrogen gas. A series of NMR spectra with parahydrogen polarization was acquired to confirm the activation of the precatalyst. A representative set of spectra is shown in Figure S9. The precatalyst activation after 20 minutes of hydrogen exposure was determined by the disappearance of the catalyst intermediate at -13.8 and -17.6 ppm. This intermediate most likely was Ir(^{Me}IMes)H_2(COD)Br.

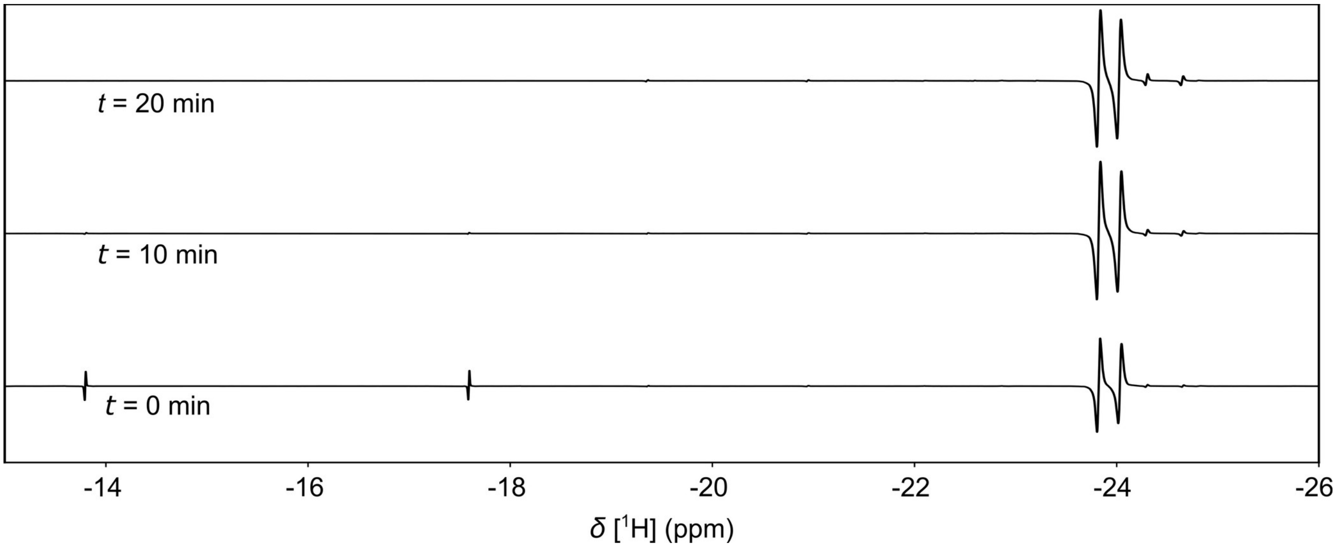

Figure S9. *Activation of a reverse-micelle sample consisting of 100 mM CTAB, 0.5 mM precatalyst, 0.1 mM ligand, 10 mM coligand, and 3% buffer faction in* $CDCl_3$*/heptane. The initial time point was a few seconds after the sample was completely pressurized with 120 psi parahydrogen.*

Effect of Protein on the Hydride Signals

A representative spectrum of the reverse micelle solution in the presence of protein is plotted in Figure S10. With the addition of protein in the reverse-micelle solution, the intensity of the complex (1) remains almost unchanged compared to Figure 2 in the main text. However, due to the additional formation of a protein-ligand complex in the presence of protein, the ligand availability reduces the concentration of complex (2), and hence, the intensity of the complex (2) at -24.3 and -24.7 ppm decreases.

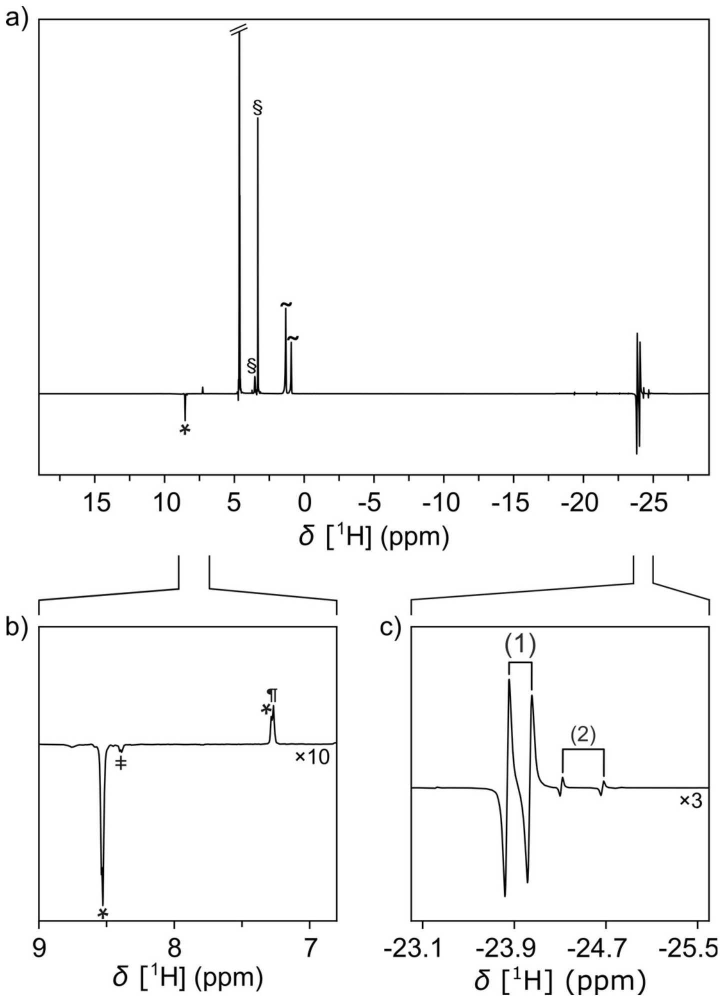

Figure S10. *a) ^{1}H spectra of reverse-micelle samples consisting of 100 mM CTAB, 0.5 mM precatalyst, 0.1 mM ligand, 10 mM coligand, and 3% buffer faction in $CDCl_3$/heptane in the presence of 0.102 mM TenA protein. The spectrum contains suppressed heptane signals at 1.35 and 0.95 ppm (~), CTAB signals at 3.54 and 3.33 ppm (§), water signal at 4.65 ppm and orthohydrogen signal at 4.62 ppm (truncated by double-dash). b) Spectral region from 9-6.8 ppm showing unbound coligand 4-tert-butylpyridine (*) and metal-bound coligand (‡). The residual $CDCl_3$ proton solvent peak is denoted by ¶. c) Spectral region from -23 to -25.6 ppm showing complex (1) and complex (2).*

Ligand Binding Experiments

The amplitudes of complex (1) and complex (2) are required to evaluate Eq. S5 in the absence and in the presence of protein. Three individual experiments were performed using three reverse micelle solutions prepared with and without protein. Once the catalyst was fully activated as indicated by the disappearance of the catalyst signal intermediate at -13.8 and -17.6 ppm, five successive spectra were acquired with parahydrogen bubbling before each acquisition. The fitted parameters for the iridium hydride signals of complex (2) at -24.3 and -24.7 ppm, and of complex (1) at -23.8 and -24.0 ppm were used for calculating the iridium hydride ratios R_H. The fitting results are plotted in Figures S11 and S12. The fit results and average R_H in the absence and presence of protein for each sample are tabulated in Tables S1 and S2.

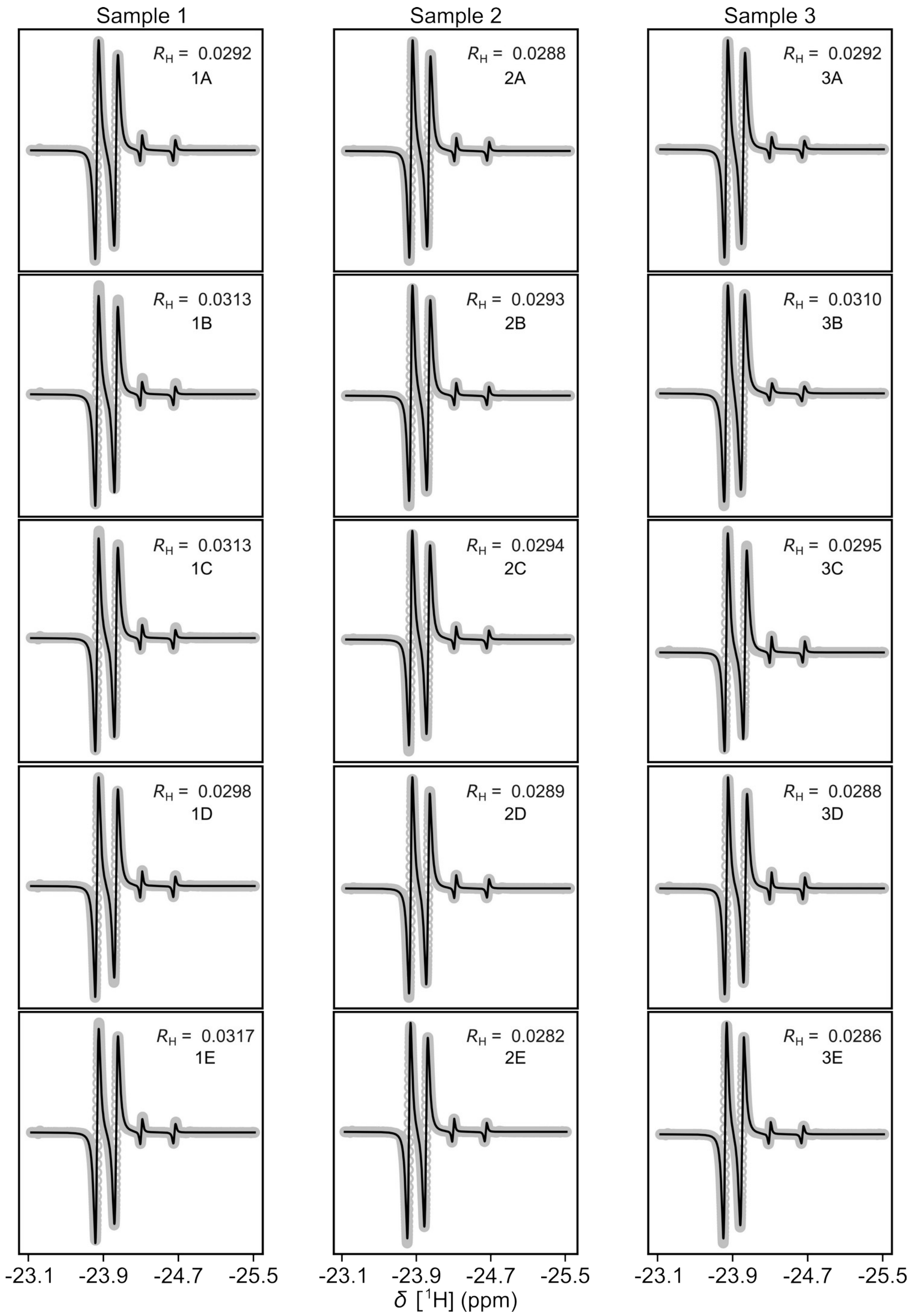

Figure S11. *Hydride region of NMR spectra containing iridium complexes (1) and (2) in reverse micelle samples without protein, recorded after introduction of parahydrogen. After complete catalyst activation, 5 successive spectra were acquired for each sample with parahydrogen bubbling for 15 s before acquisition. Data points are shown with gray circles (o), and fitted Lorentzian functions are plotted with a solid black line (-).*

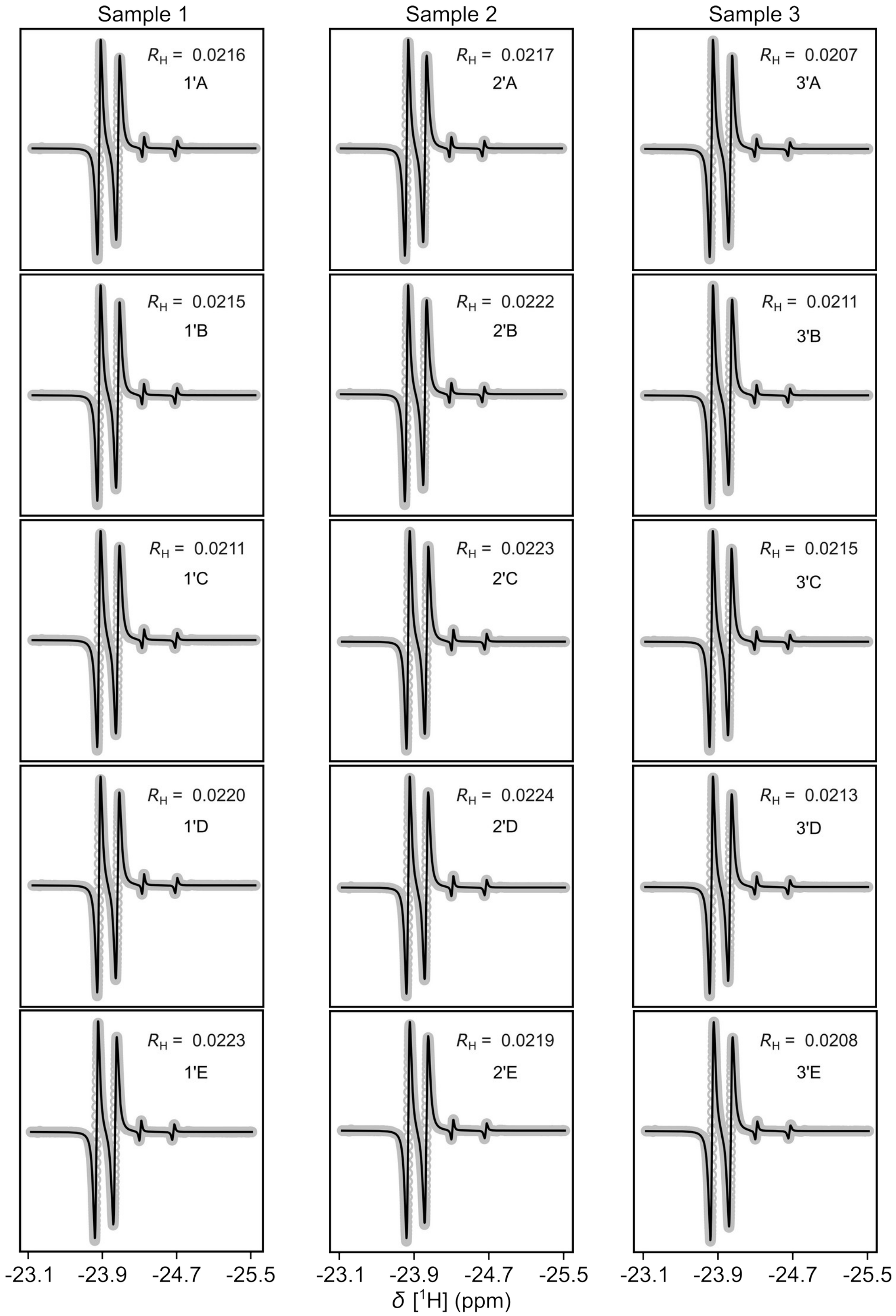

Figure S12. *Hydride region of NMR spectra containing iridium complexes (1) and (2) in reverse micelle samples with protein, recorded after introduction of parahydrogen. After complete catalyst activation, 5 successive spectra were acquired for each sample with parahydrogen bubbling for 15 s before acquisition. Data points are shown with gray circles (o), and fitted Lorentzian functions are plotted with a solid black line (-).*

Table S1. *Signal ratios obtained in the absence of TenA protein (experiments 1–3; left side). Five separate readings from each sample are denoted with the letters A–E. The standard deviations were calculated from the 5 readings for each sample.*

Exp No	Summation of Amplitude for complex (1) $A_{1,IrC2} + A_{2,IrC2}$	Summation of Amplitude for complex (2) $A_{1,IrCL} + A_{2,IrCL}$	$R_H = (A_{1,IrCL} + A_{2,IrCL})/(A_{1,IrC2} + A_{2,IrC2})$
1A	128.48	3.75	0.0292
1B	124.60	3.90	0.0313
1C	126.81	3.97	0.0313
1D	127.91	3.81	0.0298
1E	126.14	4.00	0.0317
Avg	126.8 ± 1.4	3.89 ± 0.09	$(3.07 \pm 0.10)\cdot10^{-2}$
2A	141.14	4.06	0.0288
2B	142.14	4.16	0.0293
2C	140.79	4.14	0.0294
2D	134.12	3.87	0.0289
2E	136.41	3.84	0.0282
Avg	138.9 ± 3.1	4.01 ± 0.13	$(2.89 \pm 0.04)\cdot10^{-2}$
3A	139.05	4.06	0.0292
3B	145.91	4.53	0.0310
3C	122.93	3.63	0.0295
3D	125.90	3.63	0.0288
3E	134.42	3.84	0.0286
Avg	133.64 ± 8.43	3.94 ± 0.70	$(2.94 \pm 0.09)\cdot10^{-2}$

Table S2. *Signal ratios obtained in presence of TenA protein (experiments 1'–3'). Five separate readings from each sample are denoted with the letters A–E. The standard deviations were calculated from the 5 readings for each sample.*

Exp No	Summation of Amplitude for complex (1) $A_{1,IrC2} + A_{2,IrC2}$	Summation of Amplitude for complex (2) $A_{1,IrCL} + A_{2,IrCL}$	$R_H = (A_{1,IrC2} + A_{2,IrC2})/(A_{1,IrCL} + A_{2,IrCL})$
1'A	110.52	2.38	0.0216
1'B	113.29	2.43	0.0215
1'C	109.20	2.31	0.0211
1'D	112.63	2.47	0.0220
1'E	115.29	2.57	0.0223
Avg	112.2 ± 2.1	2.43 ± 0.09	$(2.17 \pm 0.04)\cdot10^{-2}$
2'A	108.86	2.37	0.0217
2'B	111.34	2.47	0.0222
2'C	110.72	2.47	0.0223
2'D	111.56	2.50	0.0224
2'E	108.80	2.39	0.0219
Avg	110.3 ± 1.2	2.44 ± 0.05	$(2.21 \pm 0.02)\cdot10^{-2}$
3'A	125.27	2.59	0.0207
3'B	127.69	2.69	0.0211
3'C	126.98	2.72	0.0215
3'D	128.30	2.74	0.0213
3'E	127.80	2.65	0.0208
Avg	127.2 ± 1.1	2.68 ± 0.05	$(2.11 \pm 0.03)\cdot10^{-2}$

Dynamic Light Scattering Measurements

The size distribution of the reverse micelles was measured using dynamic light scattering. Measurements were performed up to 24 hr after preparation to characterize the stability of the reverse micelles. The sample contained $[Ir]_{tot}$ = 500 µM, $[L]_{tot}$ = 100 µM, $[C]_{tot}$ = 10 mM, θ_{org} = 0.97 and θ_{aq} = 0.03. The organic part comprised 450 µL of chloroform and 485 µL of heptane for 965 uL of total sample volume. A Zetasizer Nano ZS dynamic light scattering instrument was used.

The observed diameter was on the order of 10 nm in all samples (Figure S13). This diameter is comparable to the size reported for similar systems.[3]

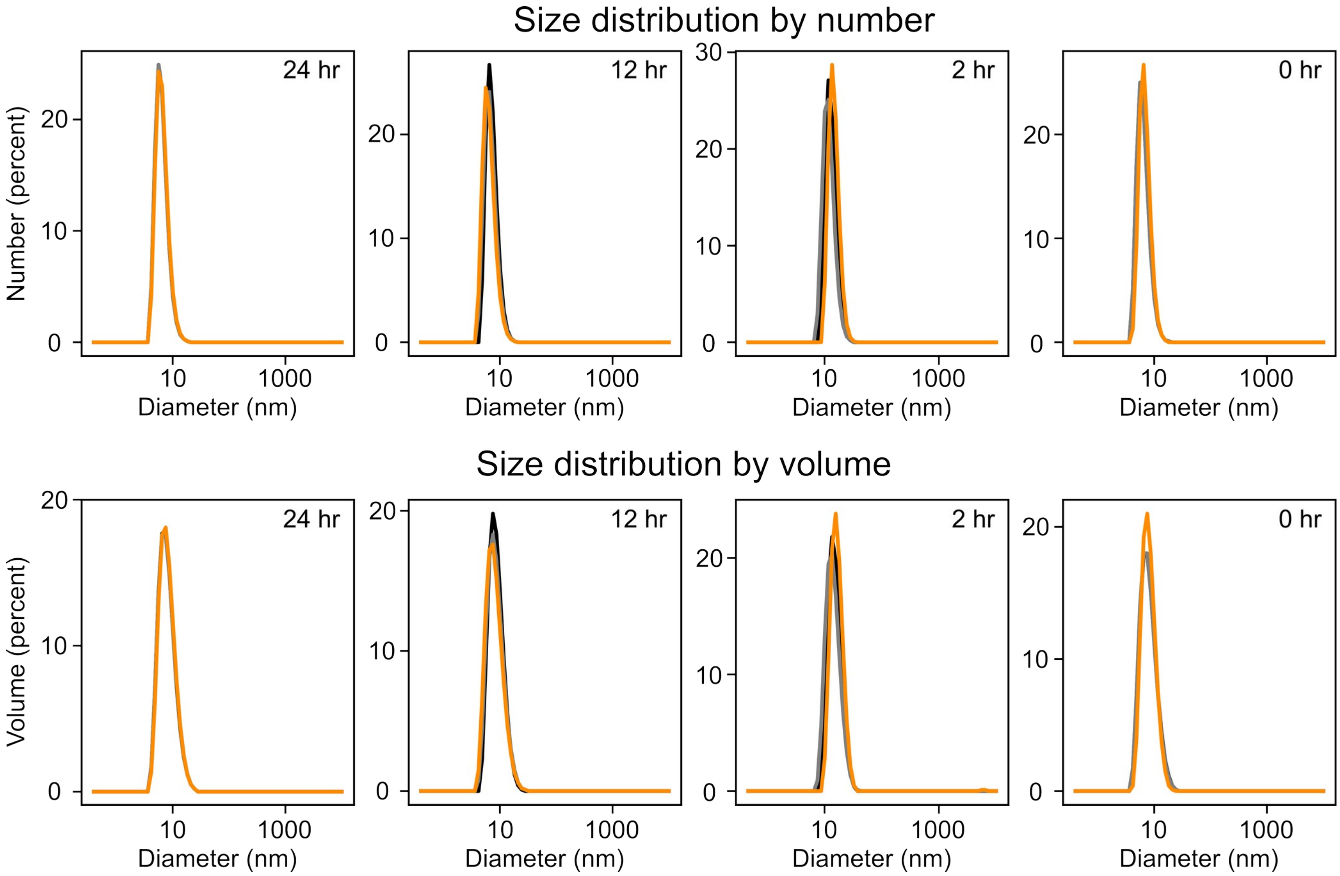

Figure S13. *The hydrodynamic diameter of the reverse micelle samples obtained through DLS analysis at 24 hr, 12 hr, 2 hr and 0 hr after sample preparation. Three data sets obtained for each sample are superimposed.*

Table S3. *The hydrodynamic diameter of the reverse micelle samples at various time periods*

Time (hr)	Z-Average (d.nm)	Polydispersity Index (PDI)	Size distribution by vol (d.nm)	Size distribution by number (d.nm)
0	10.45	0.194	8.266 ± 2.955	6.559 ± 1.802
2	10.50	0.150	8.850 ± 3.001	6.865 ± 1.950
12	10.75	0.124	8.453 ± 3.130	6.618 ± 1.856
24	10.62	0.174	8.453 ± 3.027	6.562 ± 1.810

Equilibrium Calculations

The concentrations in the reaction mixtures are modeled using a set of three equilibrium processes. These processes include the binding of the ligand to the iridium complex when exchanging a coligand for the ligand, characterized by an equilibrium constant K_{eq} (Eq. 2 in main text). Secondly, the ligand partitions between the organic and the aqueous phases with a partition coefficient K_{par} (Eq. 3). Thirdly, in the aqueous phase, the ligand binds to the protein in an equilibrium characterized by the dissociation constant K_D (Eq. 4). It is assumed that the protein is located only in the aqueous phase, and that the Ir complexes and coligand are only in the organic phase. Iridium complexes other than those listed are assumed to have insignificant concentrations.

The equilibrium constant K_{eq} for the ligand exchange in the Ir complex, the partition coefficient K_{par} for the ligand in the organic and aqueous phase, and the dissociation constant K_D of the ligand and protein are given as

$$K_{eq}=\frac{[C]_{f,org}[IrCL]_{org}}{[L]_{f,org}[IrC_2]_{org}} \quad \text{(S6)}$$

$$K_{par}=\frac{[L]_{f,aq}}{[L]_{f,org}} \quad \text{(S7)}$$

$$K_D=\frac{[P]_{f,aq}[L]_{f,aq}}{[PL]_{aq}} \quad \text{(S8)}$$

The mass balances of the participating species are

$$[Ir]_{tot}=\theta_{org}[IrCL]_{org}+\theta_{org}[IrC_2]_{org} \quad \text{(S9)}$$

$$[L]_{tot}=\theta_{org}[L]_{f,org}+\theta_{org}[IrCL]_{org}+\theta_{aq}[L]_{f,aq}+\theta_{aq}[PL]_{aq} \quad \text{(S10)}$$

$$[C]_{tot}=2\theta_{org}[IrC_2]_{org}+\theta_{org}[C]_{f,org}+\theta_{org}[IrCL]_{org} \quad \text{(S11)}$$

$$[P]_{tot}=\theta_{aq}[P]_{f,aq}+\theta_{aq}[PL]_{aq} \quad \text{(S12)}$$

The subscript tot indicates a total concentration calculated as the number of moles divided by the total sample volume. Subscripts org or aq indicate the concentrations in the aqueous or organic phase, respectively. θ_{aq} is the volume fraction of the aqueous phase and $\theta_{org} = 1 - \theta_{aq}$ of the organic phase.

The ratio R_H of the Ir hydride signals is defined in Eq. S5 and can be measured from the spectra. Here, R can be defined to be equal to the concentration ratio of complex (2) to complex (1),

$$R=\frac{[IrCL]_{org}}{[IrC_2]_{org}} \quad \text{(S13)}$$

We assume R_H is equal to R and hence will put values of experimentally obtained R_H in place of R in the equations.

Concentrations of species appearing only in the organic phase are calculated from Eq. S9

$$[IrC_2]_{org}=\frac{[Ir]_{tot}}{(1+R)\theta_{org}} \quad \text{(S14)}$$

$$[IrCL]_{org}=\frac{R[Ir]_{tot}}{(1+R)\theta_{org}} \quad \text{(S15)}$$

and from Eq. S11

$$[C]_{f,org}=\frac{(1+R)[C]_{tot}-(2+R)[Ir]_{tot}}{(1+R)\theta_{org}} \quad \text{(S16)}$$

Calculation of K_{eq} from an Experiment without Protein

In an experiment without protein, $[P]_{tot} = [P]_{f,aq} = [PL]_{aq} = 0$. The K_{eq} can be found starting from Eq. S10,

$$[L]_{f,org}=\frac{(1+R)[L]_{tot}-R[Ir]_{tot}}{(1+R)(\theta_{org}+K_{par}\theta_{aq})} \tag{S17}$$

Combining the above expressions with Eq. S6,

$$K_{eq}=\frac{(1+R)[C]_{tot}-(2+R)[Ir]_{tot}}{(1+R)[L]_{tot}-R[Ir]_{tot}}\cdot\frac{R(\theta_{org}+K_{par}\theta_{aq})}{\theta_{org}} \tag{S18}$$

Calculation of K_D from an Experiment with Protein and using K_{eq}

In an experiment with protein, the additional species P and PL are present. If K_{eq} is known from a previous experiment without protein, the K_D for the protein-ligand interaction can be calculated.

From Eqs. S6 and S7,

$$[L]_{f,org}=\frac{R[C]_{f,org}}{K_{eq}} \tag{S19}$$

$$[L]_{f,aq}=\frac{R\,K_{par}[C]_{f,org}}{K_{eq}} \tag{S20}$$

From Eq. S10,

$$[PL]_{aq}=\frac{1}{\theta_{aq}}\left([L]_{tot}-\frac{R(\theta_{org}+K_{par}\theta_{aq})[C]_{f,org}}{K_{eq}}-\frac{R[Ir]_{tot}}{1+R}\right) \tag{S21}$$

From Eq. S12,

$$[P]_{f,aq}=\frac{[P]_{tot}}{\theta_{aq}}-[PL]_{aq}=\frac{1}{\theta_{aq}}\left([P]_{tot}-[L]_{tot}+\frac{R(\theta_{org}+K_{par}\theta_{aq})[C]_{f,org}}{K_{eq}}+\frac{R[Ir]_{tot}}{1+R}\right) \tag{S22}$$

K_D is obtained by substituting the results from Eqs. S20–S22 into Eq. S8.

Experimental Values

The above equations were used to calculate the equilibrium constants in the TenA/ABAP system in reverse micelles. The reverse micelle solutions were prepared with $[Ir]_{tot}$ = 500 µM, $[L]_{tot}$ = 100 µM, $[C]_{tot}$ = 10 mM, $[P]_{tot}$ = 102.18 µM, θ_{org} = 0.97 and θ_{aq} = 0.03. The average values and errors in the resulting K_{eq} and K_D values were determined using Monte Carlo simulations using a total of 10^5 data sets. Each set was generated by randomly picking one of the three measured R_H values with protein and without protein, respectively (Tables S1 and S2), and adding a normally distributed error with the corresponding standard deviation (Figure S14 and S15). In each set, a normally distributed error with a standard deviation of 5% was added to each of the concentrations of $[Ir]_{tot}$, $[L]_{tot}$, $[C]_{tot}$, and $[P]_{tot}$ to represent the estimated concentration error. The K_{par} value of 0.216 ± 0.028 was determined experimentally from Eq. S29 as described below, and was included in the simulation with a corresponding normally distributed error. The concentration values and K_{eq} and K_D were calculated for each set of data. The mean resulting values are tabulated in Table S4. The mean K_D obtained is 39.7 ± 8.9.

Table S4. *The concentration values of different species and the equilibrium constants obtained in the absence and presence of protein TenA from Monte Carlo simulations.*

In absence of TenA protein	
$[IrC_2]_{org}$	500 ± 25 µM
$[IrCL]_{org}$	14.84 ± 0.92 µM
$[C]_{f,org}$	9282 ± 520 µM
$[L]_{f,org}$	87.7 ± 5.2 µM
K_{eq}	3.16 ± 0.29
In presence of TenA protein	
$[IrC_2]_{org}$	504 + 25 µM
$[IrCL]_{org}$	10.9 ± 0.6 µM
$[C]_{f,org}$	9288 ± 518 µM
$[L]_{f,aq}$	13.8 ± 1.2 µM
$[PL]_{aq}$	897 ± 120 µM
$[P]_{f,aq}$	2508 ± 206 µM
K_D	39.7 ± 8.9 µM

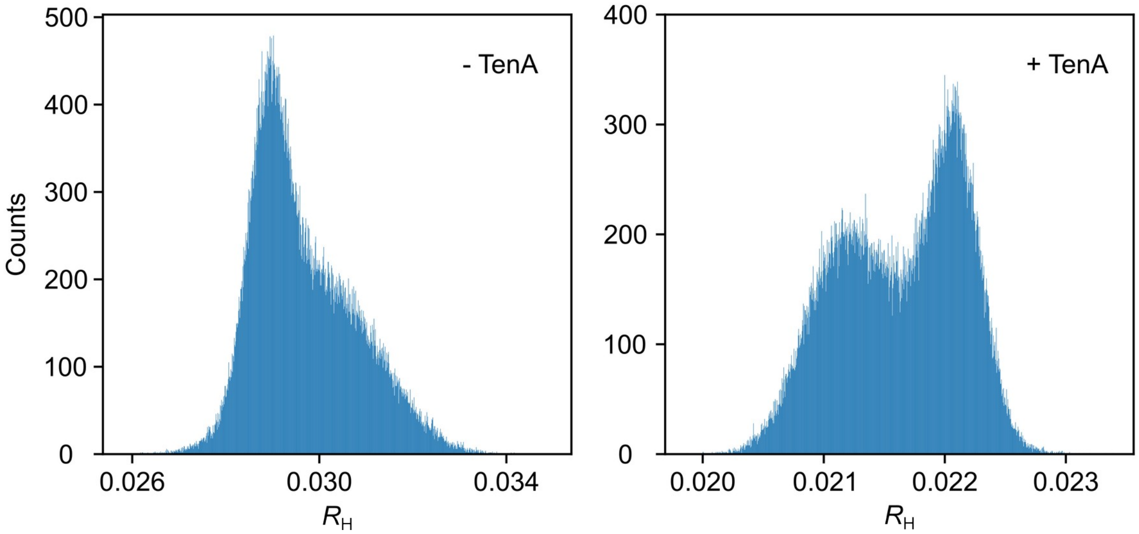

Figure S14: *Histograms showing the distributions of input values of R_H in the absence and presence of TenA protein for the Monte Carlo simulations.*

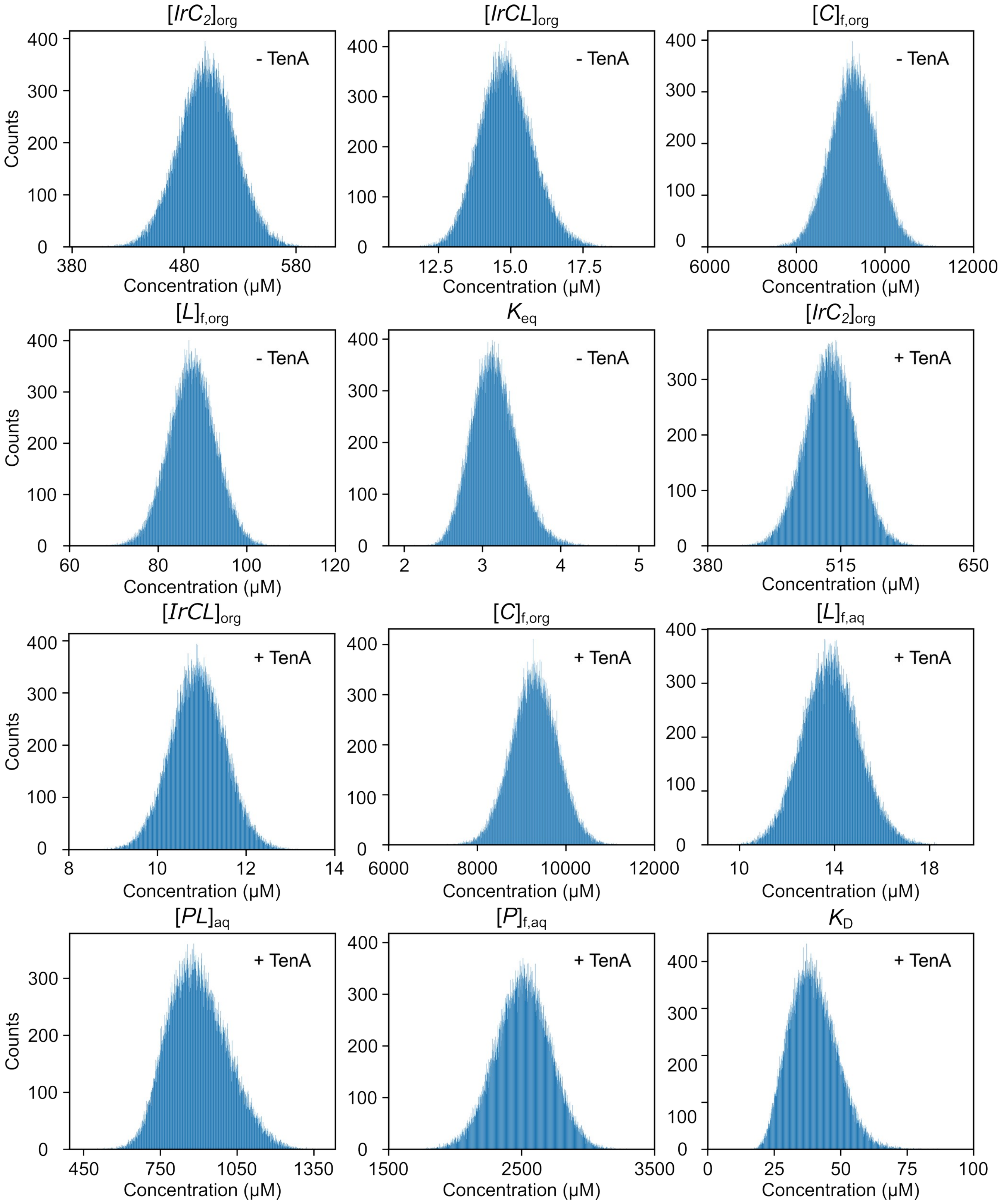

Figure S15: *Histograms showing the distributions of output values obtained from Monte Carlo simulations. The species concerned is specified in the title of each figure. The values obtained in the absence (-) and presence (+) of TenA protein are marked in the corresponding panels.*

Enzymatic Assay

An enzymatic assay was performed to determine the inhibition constant k_I of ABAP towards TenA. The assay was based on the Lineweaver-Burk equation,[4]

$$\frac{1}{v}=\frac{1}{v_{max}}+\frac{k_m}{v_{max}}\cdot\left(1+\frac{[inhibitor]}{k_1}\right)\cdot\frac{1}{[substrate]} \quad \text{(S23)}$$

The initial velocity of substrate turnover is proportional to the initial rate of change of the absorbance and the reciprocal of the difference of the molar extinction coefficients of the substrate and reaction product,

$$v\propto\frac{1}{\epsilon_{product}-\epsilon_{substrate}}\cdot\frac{\Delta A}{\Delta t} \quad \text{(S24)}$$

Here, $\epsilon_{product}$ and $\epsilon_{substrate}$ are the molar extinction coefficients of the product and substrate, respectively. $\Delta A/\Delta t$ is the rate of change of the measured absorbance.

Thiamine was used as the substrate for the assay. The UV spectra of thiamine and TenA are shown in Figure S16. The change of absorbance at 268 nm was used as the parameter for determining the kinetics of this enzyme. This wavelength as a local absorbance maximum of thiamine was chosen to minimize the measurement deviation, and the absorbance of 2 µM TenA at this wavelength did not significantly contribute to the observed absorbance. Even though the spectra of products were not available for comparison to determine the optimal wavelength for absorbance change, the change at 268 nm was sufficient for conducting the enzymatic assay of TenA.

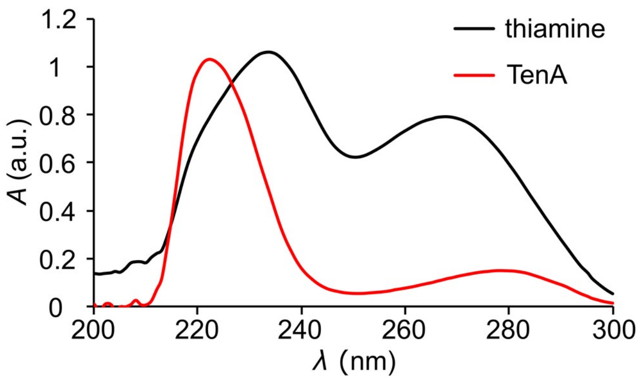

Figure S16: *UV spectra of 100 µM thiamine and 2 µM TenA.*

Kinetic experiments were conducted to determine the initial rate of change of absorbance, $\Delta A/\Delta t$, in the presence and absence of ABAP. The initial thiamine concentrations were 30, 60, and 90 µM, and the concentration of TenA was 2 µM. For inhibition experiments, the concentration of ABAP, as the inhibitor, was fixed at 50 µM. The temperature was at 23 °C. Three trials were conducted for each concentration set, and the average enzymatic rates are plotted in Figure S17.

The Lineweaver-Burk equation was fitted to the data sets with and without inhibition by ABAP. For fitting, a linear regression method was coded in the Python language (version 3.10, Python Software Foundation, Wilmington, DE). The two fitted lines converge at the same y-intercept, which indicates

that ABAP is a competitive inhibitor that binds to the active site of TenA. The inhibition constant is determined from the slopes m of the fitted lines as K_I = [inhibitor]/($m_{inhibited}$/$m_{non\text{-}inhibited}$ – 1) = 39.8 ± 6.9 µM.

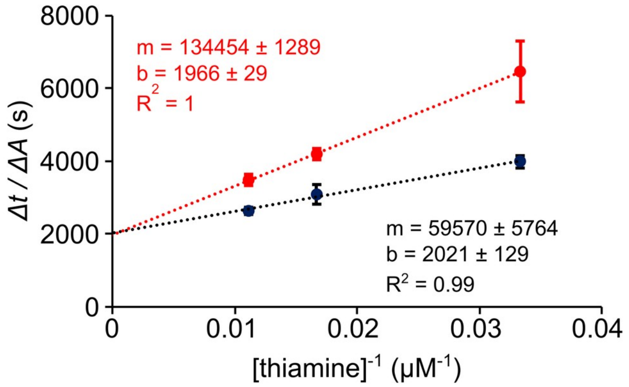

Figure S17: *Lineweaver-Burk plot of TenA kinetics. The experiments used three initial thiamine concentrations of 30, 60, and 90 µM, 2 µM TenA. 50 µM of ABAP was included for only the inhibition experiments. Each data point represents an averaged initial rate of thiamine consumption at 23 °C from 3 measurements. The kinetics of the non-inhibition experiments and inhibition experiments are plotted in black (smaller slope) and red (larger slope), respectively.*

Simulation of Change in R_H values

In order to assess experimental parameter values that result in an observable change in the R_H values, parameters for the nhPHIP method, simulations of the binding equilibria were performed. In figure S18, the ratio of R_H with and without protein,

$$RR_H = \frac{(R_H)_{+TenA}}{(R_H)_{-TenA}} \qquad \text{(S25)}$$

is shown. In Figure S18, K_{par} ranges from 10^{-3}–10^{3}, while K_D spans from 1 nM–100 mM, keeping all the other parameters at their experimental values. Simulated values can provide general guidance for the design of experiments. From the figure it can be seen that ligands having lower K_D value (stronger binding to protein) show a larger change in R_H when having higher hydrophobicity (lower K_{par}) under conditions that are otherwise the same as in the experiments.

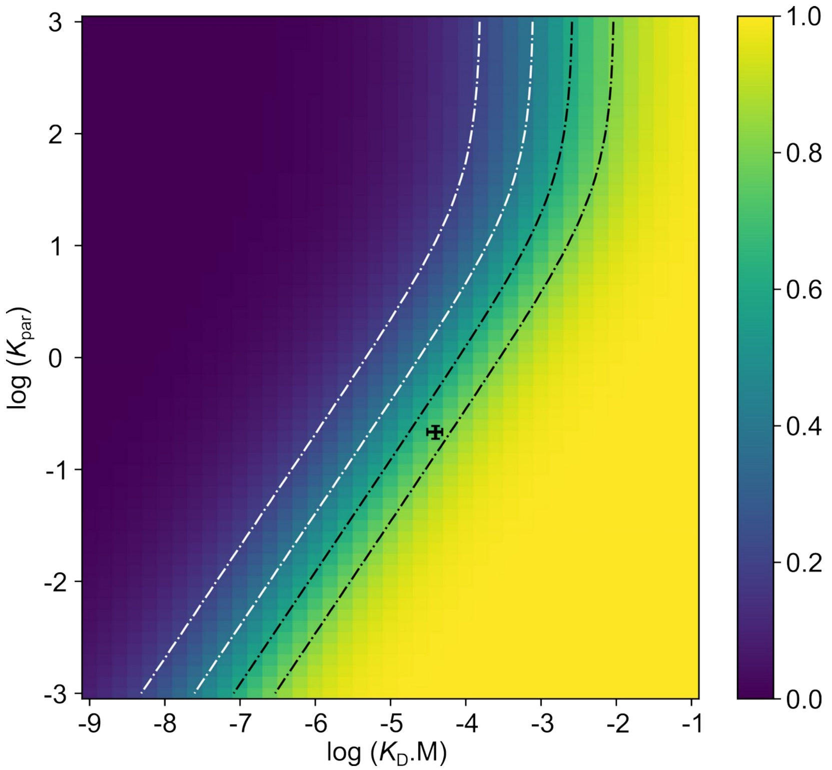

Figure S18. *A simulation for the calculated relative ratios RR_H of the R_H values between the experiments in the presence and absence of TenA protein. For the simulation, the total concentrations were set at 100 µM for the ligand, 500 µM for [IrCl(COD)(^{Me}IMes)], and 10,000 µM for the coligand. The total concentration of protein is 102 µM for experiments with proteins. The other fixed parameters include K_{eq} = 3.16 and θ_{aq} = 3%. K_{par} values vary from 10^{-3} to 10^3, while the K_D values range from 1 nM to 100 mM. The experimental values of the ligand for TenA in this work are indicated with error bars, K_{par} = 0.216 ± 0.028 and K_D = 39.7 ± 8.9 µM. The corresponding RR_H value is 0.734. The RR_H values are contoured with 0.2 increments by dashed-dotted curves.*

Dependence of Hydride Signal Intensity on Ligand Concentration

In the following experiment, we evaluate how the ratio of complex (2) to complex (1) changes with respect to ligand concentration. The precatalysts were activated in the absence of protein according to the previously described procedure. Parahydrogen gas at 120 psi was bubbled through the sample with a flow rate of 0.2 standard liters per minute for 15 seconds and stabilized for 4 seconds before applying NMR pulses. Four concentrations of ligands were used for four experiments (400 µM, 200 µM, 100 µM, and 50 µM), keeping the coligand concentration the same in all cases (10 mM), and the R_H was evaluated using Eq. S5. A total of 3 sets of experiments were performed for each concentration of ligand to check the reproducibility of the experiments (Figure S19). Lorentzian functions were fitted to the spectral lines, as described in the data processing section. The fitted parameters (amplitude of the hydride signals of complex (1) and (2) for the iridium complex (2) (-24.3 and -24.7 ppm) and of complex (1) (-23.8 and -24.0 ppm) were used for obtaining the fitted curves and calculating the hydride ratios R_H, as shown in the Figure S19. The fitted amplitudes obtained from Figure S19 are tabulated in Table S5 below. The hydride ratios R_H for each ligand concentration and the mean values are tabulated in Table S6 below.

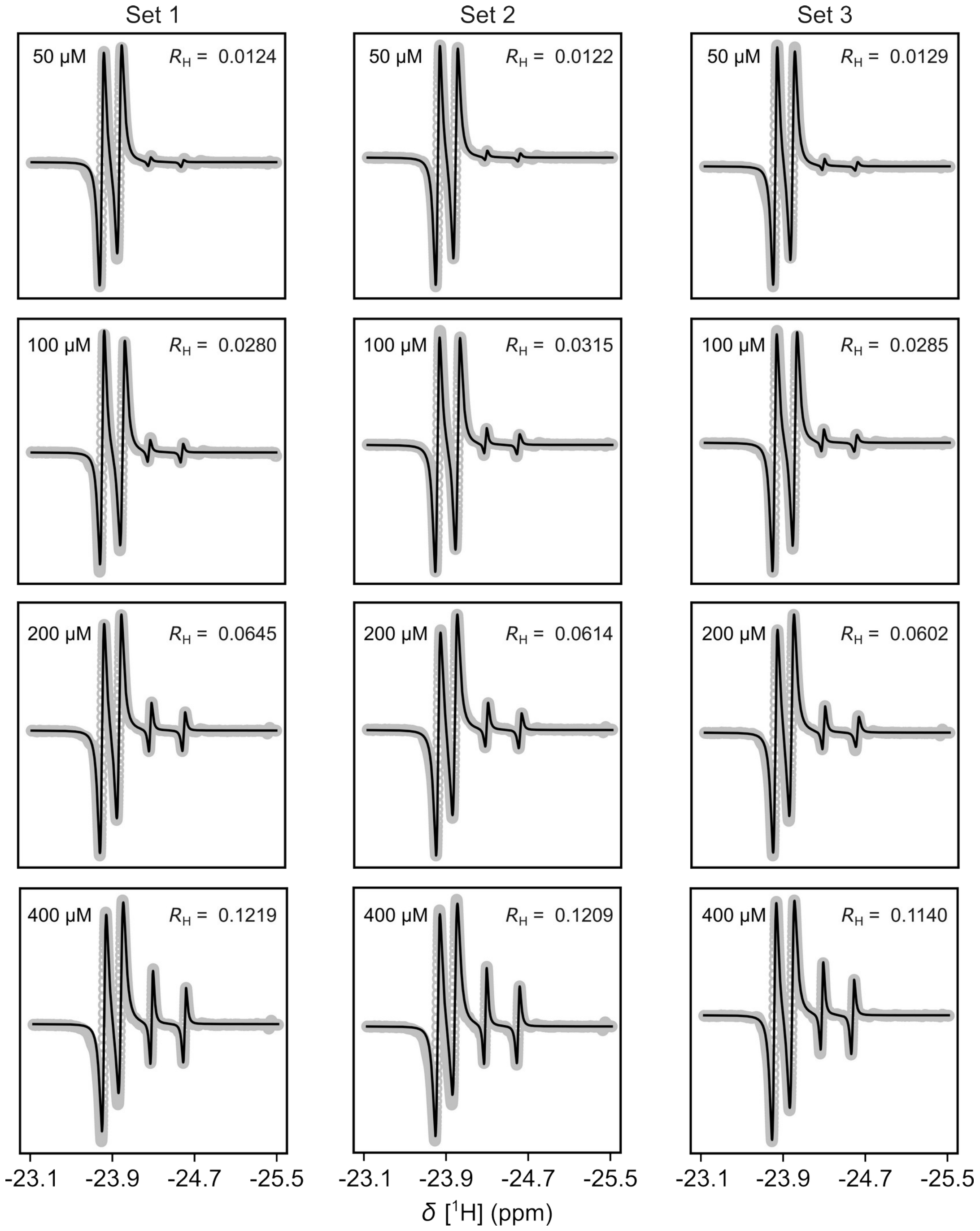

Figure S19. *Hydride region of NMR spectra containing iridium complexes (2) and (1) in reverse micelle samples without protein, containing different concentrations of ligand (50 μM, 100 μM, 200 μM, and 400 μM). The spectra were recorded after introduction of parahydrogen.*

Table S5. *The experimental parameters for complex (1) and complex (2).* $A_{1,\mathrm{IrCL}}$ $A_{2,\mathrm{IrCL}}$, $A_{1,\mathrm{IrC2}}$, *and* $A_{2,\mathrm{IrC2}}$ *are the fitted amplitudes obtained from Lorenztian fit.*

Concentration /µM	Summation of Amplitude for complex (1) ($A_{1,\mathrm{IrCL}} + A_{2,\mathrm{IrCL}}$)				Summation of Amplitude for complex (2) ($A_{1,\mathrm{IrC2}} + A_{2,\mathrm{IrC2}}$)			
	Set 1	Set 2	Set 3	Average	Set 1	Set 2	Set 3	Average
50	17.35	13.10	13.60	14.7 ± 1.9	0.22	0.16	0.17	0.18 ± 0.02
100	16.11	11.02	14.93	14.0 ± 2.2	0.45	0.35	0.43	0.41 ± 0.04
200	12.98	12.33	12.23	12.5 ± 0.3	0.84	0.76	0.74	0.78 ± 0.04
400	10.22	11.99	12.25	11.5 ± 0.9	1.25	1.45	1.40	1.36 ± 0.09

Table S6. *Calculated* R_H *value from amplitude tabulated in Table S5.*

Concentration/µM	$R_\mathrm{H} = (A_{1,\mathrm{IrC2}} + A_{2,\mathrm{IrC2}})/(A_{1,\mathrm{IrCL}} + A_{2,\mathrm{IrCL}})$			
	Set 1	Set 2	Set 3	Average
50	0.0124	0.0122	0.0129	$(1.25 \pm 0.03)\cdot 10^{-2}$
100	0.0280	0.0315	0.0285	$(2.93 \pm 0.16)\cdot 10^{-2}$
200	0.0645	0.0614	0.0602	$(6.20 \pm 0.18)\cdot 10^{-2}$
400	0.1219	0.1209	0.1140	$(11.90 \pm 0.35)\cdot 10^{-2}$

The standard deviation obtained for the R_H values for each concentration arises from the three sets of experiments conducted for each concentration. The error obtained was up to ~5%.

The K_D was calculated using the hydride ratio R_H of complex (1) and complex (2) instead of the amplitude of complex (2) alone. The error obtained for the amplitude of complex (1) for three different samples was 15% or less, and for complex (2), it was 13% or less. In contrast, the maximum error obtained for the calculation of the hydride ratio R_H for the three samples was only ~5%.

The dependence of R on the ligand concentration is obtained by solving Eq. S18,

$$R = -[C]_{tot} f + 2f[Ir]_{tot} - [Ir]_{tot} K_{eq} + K_{eq}[L]_{tot} \pm \frac{\sqrt{a}}{2f([C]_{tot} - [Ir]_{tot})} \tag{S26}$$

where a and f are

$$\begin{aligned} a = {} & [C]_{tot}^2 f^2 - 4[C]_{tot} f^2 [Ir]_{tot} + 2[C]_{tot} f K_{eq} [Ir]_{tot} + 2[C]_{tot} f K_{eq} [L]_{tot} + 4 f^2 [Ir]_{tot}^2 \\ & - 4 f [Ir]_{tot}^2 K_{eq} + [Ir]_{tot}^2 K_{eq}^2 - 2[Ir]_{tot} K_{eq}^2 [L]_{tot} + K_{eq}^2 [L]_{tot}^2 \end{aligned} \tag{S27}$$

$$f = \frac{\theta_{org} + K_{eq} \cdot \theta_{aq}}{\theta_{org}} \tag{S28}$$

By fitting Eq. S26, with K_{eq} as the fit parameter, the solid line shown in Figure S20 is obtained. A close agreement of the fitted line with the values of R_H for each ligand concentration, $[L]_{tot}$ is observed. This agreement further corroborates that the ratio of the iridium hydride signals, R, can be used for the concentration ratio of the corresponding complexes of IrCL (complex 2) and IrC_2 (complex 1) in the analysis. Additionally, the $K_{eq} = 3.15 \pm 0.05$ obtained from the fitted equation is in agreement with the K_{eq} values obtained in Table S4. The stated error range is from the fit, without considering a concentration error in the calculation.

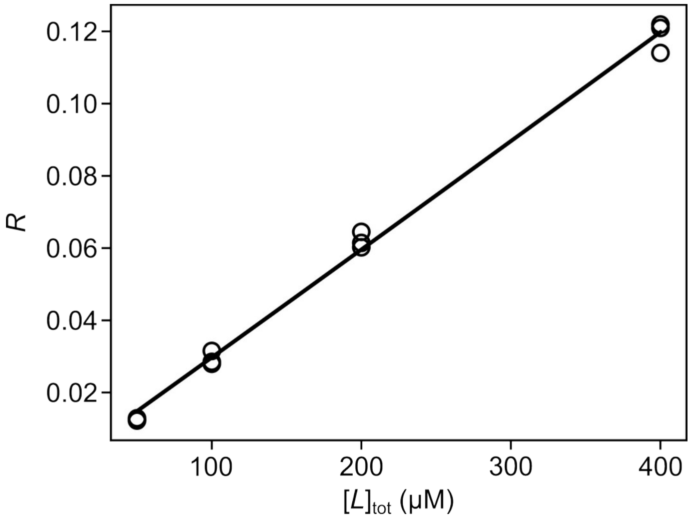

Figure S20. *Experimental values of R_H (circles) and plot of R vs. total ligand concentration fitted using Eq. S26 (solid line). K_{eq} was the fitting parameter, resulting in a value of 3.15 ± 0.05.*

Detection Limit

To evaluate the limit of detection for the current experiments, the concentration of the sensor and the ligand were lowered. Resulting spectra are shown in Figure S21.

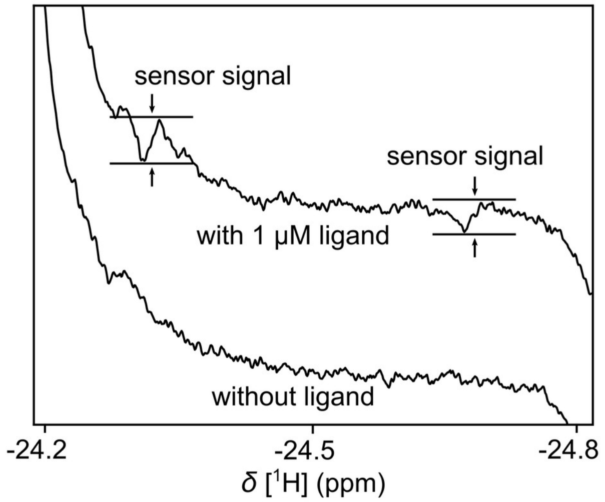

Figure S21. *NMR spectra of reverse micelle suspension containing iridium complex recorded with parahydrogen hyperpolarization in the presence (top) and absence (bottom) of 1 μM ABAP ligand. The region shown encompasses the hydride signal of the sensor with ligand ([complex (2)] = 0.12 μM),*

showing SNR = 11.4 for the signal at -24.0 ppm. The foot of the larger signal of the sensor that does not contain the ligand ([complex (1)] = 499.88 μM) is visible to the left of the range. For the experiments, the precatalysts were activated as described above.

Determination of Organic/Aqueous Partition Coefficient K_{par}

The partition coefficient of ABAP in a solvent mixture of $CDCl_3$/heptane (50/50 v/v) and water solution was determined. An organic solution of 1 mM ABAP was prepared in $CDCl_3$/heptane (50/50 v/v). For each preparation, 1 mL of 50 mM tris buffer with pH of 8.0 was added to 1 mL of 4-amino-2-benzylaminopyrimidine solution. These heterogeneous samples were vortexed for 1 minute and then centrifuged at 5000 g for 5 minutes. The aqueous portions were discarded, and the organic portions were collected. Four preparations and the original solution were analyzed by NMR. The signal integrals of all aromatic protons were used to determine the partition coefficient

$$K_{par} = \frac{S_{orig} - S_{prep}}{S_{prep}} \tag{S29}$$

S_{orig} and S_{prep} are the signal integrations of the original solution and the preparations, respectively. The partition coefficient value was averaged from four trials as $K_{par} = 0.216 \pm 0.028$.

Protein Expression and Purification

The expression and purification of TenA were previously described.[5] The procedure with any modifications is summarized in the following. A BL21(DE3) strain of *Escherichia coli* containing a pDESTF1 plasmid with the TenA gene was first grown overnight at 37 °C on an agar plate that was made from LB medium with 100 μg/mL ampicillin. A colony from this agar plate was inoculated to 300 mL of an LB medium with 100 μg/mL ampicillin, which was continuously shaken at 200 rpm and incubated at 37 °C overnight. A 50 mL volume of this starter culture was transferred to 0.95 L of LB medium with 100 μg/mL ampicillin, which was continuously shaken at 200 rpm and incubated at 37 °C for several hours. When the cell density reached OD_{600} of about 0.6, isopropyl β-D-thiogalactoside (IPTG) was added to a final concentration of 1 mM. After 8 hours of induction, the cells were centrifuged and stored at -80 °C until the purification step.

The frozen cells were resuspended in a binding buffer (5 mM imidazole, 50 mM sodium phosphate, 300 mM sodium chloride, pH 8.0) and lysed by sonication. Subsequently, the cell fragments were centrifuged at 15,000 g for 2 hours. The supernatant was loaded in a pre-conditioned 5 mL His-tag column (Cytiva, Histrap™ HP, Marlborough, MA). The impurities were removed by washing with 50 mL of the binding buffer and then 50 mL of a wash buffer (50 mM imidazole, 50 mM sodium phosphate, 300 mM sodium chloride, pH 8.0). TenA was eluted with an elution buffer (250 mM imidazole, 50 mM sodium phosphate, 300 mM sodium chloride, pH 8.0). The elution buffer was exchanged to a tris buffer (50 mM tris, pH 8.0) and concentrated in a centrifugal filter device using a 3000 kDa cutoff membrane (Amicon® Ultra 15 mL Centrifugal Filters, MilliporeSigma, Burlington, MA). The purity of TenA was determined using Coomassie-stained SDS-PAGE analysis. No other protein impurities were detected. The concentration of the final protein solution was determined by UV

spectrophotometry[5] (ε_{280} = 74.28 mM^{-1} cm^{-1}). A final stock solution of TenA at a concentration of 3.4 mM, was stored at 4 °C and used within 2 days.

References

(1) Rayner, P. J.; Gillions, J. P.; Hannibal, V. D.; John, R. O.; Duckett, S. B. Hyperpolarisation of Weakly Binding N-Heterocycles Using Signal Amplification by Reversible Exchange. *Chem. Sci.* **2021**, *12* (16), 5910–5917.

(2) Bengs, C.; Dagys, L.; Levitt, M. H. Robust Transformation of Singlet Order into Heteronuclear Magnetisation over an Extended Coupling Range. *J. Magn. Reson.* **2020**, *321*, 106850.

(3) Lang, Jacques.; Mascolo, Giuseppe.; Zana, Raoul.; Luisi, P. Luigi. Structure and Dynamics of Cetyltrimethylammonium Bromide Water-in-Oil Microemulsions. *J. Phys. Chem.* **1990**, *94* (7), 3069–3074.

(4) Cantor, C. R.; Schimmel, P. R. *Biophysical Chemistry; Part III: The behavior of biological macromolecules*; W. H. Freeman and Company, 1980; p 897.

(5) Toms, A. V.; Haas, A. L.; Park, J.-H.; Begley, T. P.; Ealick, S. E. Structural Characterization of the Regulatory Proteins TenA and TenI from *Bacillus subtilis* and Identification of TenA as a Thiaminase II. *Biochemistry* **2005**, *44* (7), 2319–2329.
